# Coxiella burnetii Sterol-Modifying Protein Stmp1 Regulates Cholesterol in the Intracellular Niche

**DOI:** 10.1128/mbio.03073-21

**Published:** 2022-01-25

**Authors:** Tatiana M. Clemente, Rochelle Ratnayake, Dhritiman Samanta, Leonardo Augusto, Paul A. Beare, Robert A. Heinzen, Stacey D. Gilk

**Affiliations:** a Department of Pathology and Microbiology, University of Nebraska Medical Center, Omaha, Nebraska, USA; b Department of Microbiology and Immunology, Indiana University School of Medicine, Indianapolis, Indiana, USA; c Department of Microbiology and Immunology, Midwestern University, Glendale, Arizona, USA; d Laboratory of Bacteriology, Rocky Mountain Laboratories, National Institute of Allergy and Infectious Diseases, National Institutes of Health, Hamilton, Montana, USA; University of Michigan-Ann Arbor

**Keywords:** *Coxiella*, cholesterol, intracellular pathogen, vacuoles, vesicular trafficking

## Abstract

Coxiella burnetii replicates in a phagolysosome-like vacuole called the *Coxiella*-containing vacuole (CCV). While host cholesterol readily traffics to the CCV, cholesterol accumulation leads to CCV acidification and bacterial death. Thus, bacterial regulation of CCV cholesterol content is essential for *Coxiella* pathogenesis. *Coxiella* expresses a sterol-modifying protein, Stmp1, that may function to lower CCV cholesterol through enzymatic modification. Using an Stmp1 knockout (Δ*stmp1*), we determined that Stmp1 is not essential for axenic growth. Inside host cells, however, Δ*stmp1* mutant bacteria form smaller CCVs which accumulate cholesterol, preferentially fuse with lysosomes, and become more acidic, correlating with a significant growth defect. However, in cholesterol-free cells, Δ*stmp1* mutant bacteria grow similarly to wild-type bacteria but are hypersensitive to cholesterol supplementation. To better understand the underlying mechanism behind the Δ*stmp1* mutant phenotype, we performed sterol profiling. Surprisingly, we found that Δ*stmp1* mutant-infected macrophages accumulated the potent cholesterol homeostasis regulator 25-hydroxycholesterol (25-HC). We next determined whether dysregulated 25-HC alters *Coxiella* infection by treating wild-type *Coxiella*-infected cells with 25-HC. Similar to the Δ*stmp1* mutant phenotype, 25-HC increased CCV proteolytic activity and inhibited bacterial growth. Collectively, these data indicate that Stmp1 alters host cholesterol metabolism and is essential to establish a mature CCV which supports *Coxiella* growth.

## INTRODUCTION

The obligate intracellular Gram-negative bacterium Coxiella burnetii is the causative agent of human Q fever. In its acute stage, Q fever presents with mild flu-like symptoms. However, the infection can resurface years later as debilitating fatigue or manifest as endocarditis, which is usually fatal if untreated (1). Chronic disease requires 18 to 24 months of doxycycline in combination with hydroxychloroquine, and there are currently no licensed vaccines for Q fever in the United States (2, 3). Thus, there is an urgent need to better understand C. burnetii pathogenesis and identify new virulence factors that could be used as drug targets.

Mainly transmitted by aerosols, C. burnetii first infects alveolar macrophages and directs the biogenesis of a phagolysosome-like compartment known as the *Coxiella*-containing vacuole (CCV), which is essential for bacterial replication (4, 5). The C. burnetii type 4B secretion system (T4BSS) secretes effector proteins into the host cell cytosol in order to establish and maintain CCV fusogenicity with host endocytic vesicles, a process critical for CCV expansion and subsequent bacterial replication (6–9). The promiscuous fusogenicity of the CCV with early and late endosomes, lysosomes, and autophagosomes delivers cholesterol to the CCV membrane (10, 11). Cholesterol is a major cellular lipid that regulates membrane fluidity, trafficking, and signaling in mammalian cells (12). Many intracellular pathogens target mammalian cholesterol for host cell entry, to obtain nutrients, and/or to manipulate cellular signaling (13, 14). For C. burnetii, cholesterol plays an important role during host cell infection. C. burnetii host cell entry utilizes α_V_β_3_ integrin, a transmembrane protein present in cholesterol-rich plasma membrane microdomains known as lipid rafts (15, 16). Additionally, during infection of monocytes, C. burnetii regulates expression of several host cell genes involved in cholesterol storage and efflux (17–19). Finally, the CCV membrane is sterol-rich, and treatment of C. burnetii-infected cells with drugs that perturb host cell cholesterol homeostasis also inhibits bacterial growth (20, 21). Collectively, these data had suggested that cholesterol is essential for CCV formation and C. burnetii replication. However, our cholesterol-free tissue culture model revealed that cholesterol is not required for C. burnetii growth (16). In fact, we discovered that high cholesterol levels on the CCV membrane lead to decreased fusogenicity and increased acidification, which causes bacterial degradation (10). These surprising findings indicate that elevated CCV cholesterol is toxic to C. burnetii, and the bacteria must actively modulate host cholesterol metabolism to decrease CCV cholesterol levels.

C. burnetii expresses two eukaryote-like sterol reductases, CBU1158 and CBU1206, with homology to Δ7 and Δ24 sterol reductases, respectively (22). As C. burnetii lacks the remaining enzymes in the cholesterol biosynthetic pathway, CBU1158 and CBU1206 are considered “orphan enzymes” that were likely obtained through horizontal transfer from amoebas (23–25). We previously showed that CBU1206 is an active enzyme in yeast and can generate ergosterol from ergosterol precursors (25). As this suggested that CBU1206 may have broad substrate specificity, we named it sterol-modifying protein 1 (Stmp1). Interestingly, we have not detected any evidence that Stmp1 generates cholesterol during C. burnetii infection (16). Here, we utilize an Stmp1 knockout (Δ*stmp1*) to further investigate the role of Stmp1 during C. burnetii host cell infection. Our data demonstrate that the absence of Stmp1 leads to a severe intracellular growth defect, higher fusogenicity with lysosomes, increased acidification of the CCV lumen, accumulation of cholesterol on the CCV membrane, and elevated levels of 25-hydroxycholesterol in infected cells. These data support our previous findings that C. burnetii is exquisitely sensitive to cholesterol and that regulation of cholesterol levels on the CCV membrane plays a key role in C. burnetii intracellular growth.

## RESULTS

### Stmp1 is a bacterial outer membrane protein required for C. burnetii intracellular growth and normal CCV formation.

**FIG 1:**
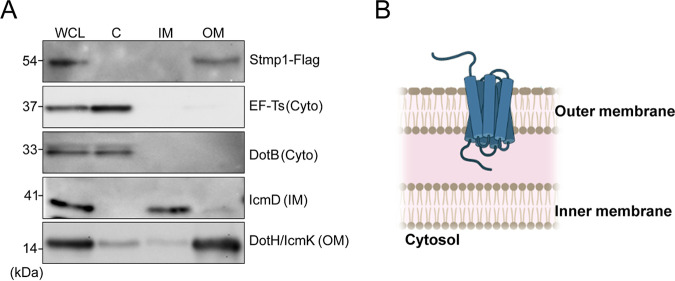
Stmp1 localizes to the bacterial outer membrane. (A) Stmp1 localization within the bacterial cell was determined by fractionation of Stmp1-Flag-expressing C. burnetii. Controls for the subcellular fractions were elongation factor Ts (EF-Ts) and DotB (cytoplasm), IcmD (inner membrane), and DotH/IcmK (outer membrane). Immunoblots blots are representative of three independent experiments. (B) Schematic representation of Stmp1 in C. burnetii.

Our previous studies demonstrated that C. burnetii expresses two homologs of eukaryotic sterol reductases, CBU1158 and CBU1206 (22). By heterologous expression, we found that CBU1206 is an active enzyme capable of binding and modifying yeast sterols (25); thus, we named this protein Stmp1 for sterol-modifying protein 1. Stmp1 contains 10 predicted transmembrane domains and is not secreted through the C. burnetii T4BSS (26), suggesting that Stmp1 is associated with the bacterial cell envelope. To determine Stmp1 localization within the bacteria, C. burnetii bacteria expressing C-terminal 3xFlag (Stmp1-Flag) were fractionated (27). Proteins found in the bacterial cytoplasm (elongation factor-Ts and ATPase dotB), inner membrane (IcmD), and outer membrane (DotH/IcmK) validated the fractionation protocol (28, 29). Stmp1-Flag was detected in the outer membrane fraction, indicating that Stmp1 localizes to the outer membrane of the bacterial cell envelope (Fig. 1A and B).

**FIG 2:**
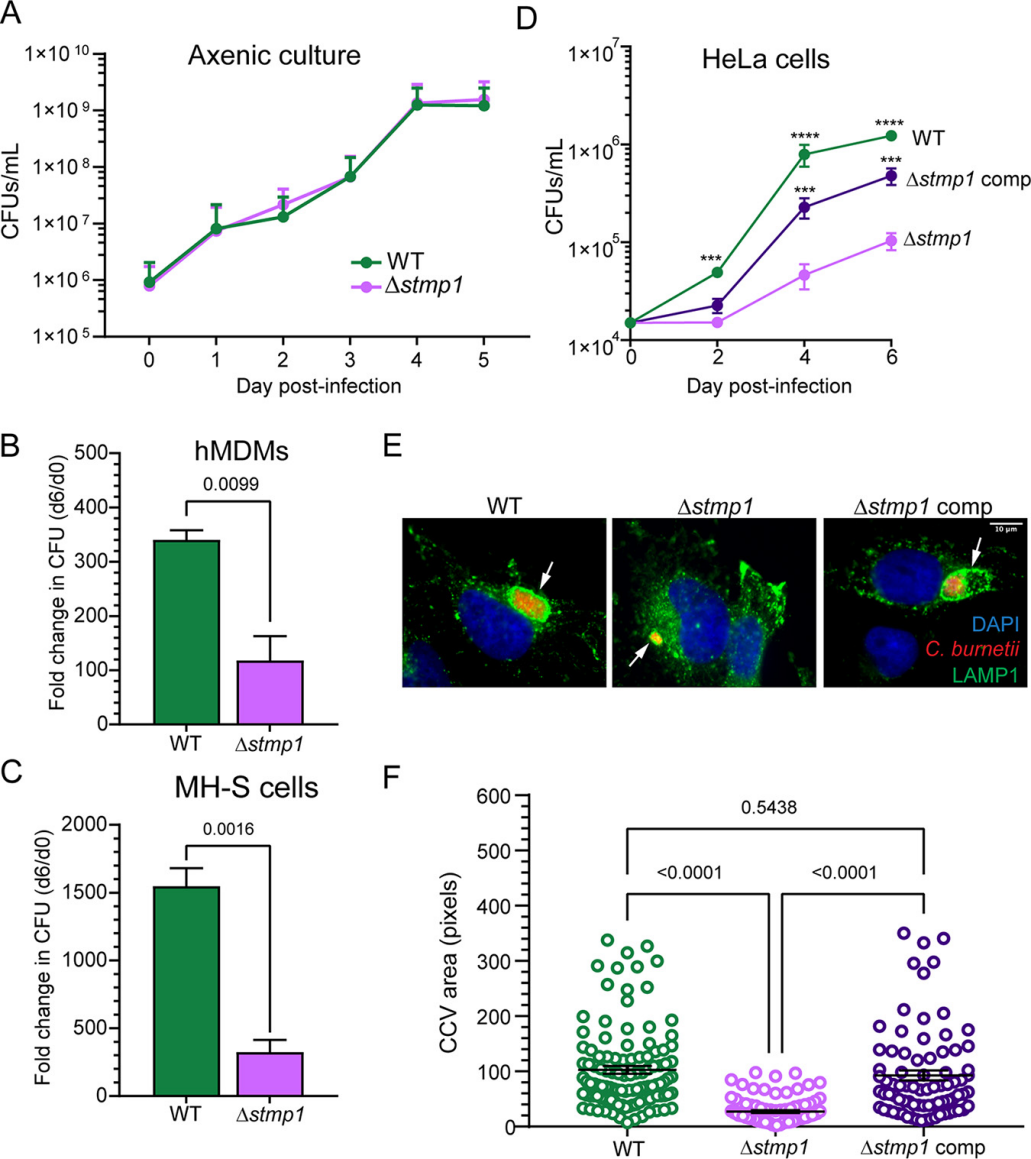
The C. burnetii Δ*stmp1* mutant has a growth defect in macrophages and epithelial cells. (A) In axenic cultures, C. burnetii WT and Δ*stmp1* mutant bacteria have identical growth over 5 days, as determined by CFU assay for bacterial numbers. (B to D) However, the C. burnetii Δ*stmp1* mutant has a significant intracellular growth defect in (B) human monocyte-derived macrophages (hMDMs), (C) murine alveolar macrophages (MH-S), and (D) HeLa cells, as determined by CFU assay. Growth was calculated as the fold change over day 0 and shown as the mean ± standard error of the mean (SEM) from three separate growth experiments done in duplicate. Statistical significance was determined by multiple *t* tests or *t* test; ***, *P* < 0.005; ****, *P* < 0.001. (E and F) Immunofluorescence staining (E) and quantitative measurements (F) indicate that Δ*stmp1* CCVs are significantly smaller than WT or complemented Δ*stmp1* CCVs. Representative images of CCVs (arrows) stained by immunofluorescence at 6 dpi. Blue, DAPI (host cell nuclei); red, C. burnetii; green, LAMP1 (lysosomes and CCV). CCV size was measured using ImageJ, with each circle representing an individual CCV. Data are shown as the mean ± SEM of at least 20 cells in each of three independent experiments. Statistical significance was determined by one-way ANOVA with Tukey’s *post hoc* test.

Given that cholesterol accumulation on the CCV membrane leads to C. burnetii death (10), we hypothesized that Stmp1 modifies host sterols in order to promote C. burnetii intracellular growth. To elucidate the role of Stmp1 during C. burnetii infection, we generated a targeted deletion mutant in C. burnetii (Δ*stmp1*) (Fig. S1A). In broth media, the Δ*stmp1* mutant grows identically to wild-type (WT) C. burnetii (Fig. 2A). We next assessed intracellular growth of the Δ*stmp1* mutant in human monocyte-derived macrophages (hMDMs), murine alveolar macrophages (MH-S), and HeLa epithelial cells using a CFU assay (30). Over 6 days, Δ*stmp1* mutant growth was significantly decreased compared to WT bacteria in hMDMs (65.3% decrease, Fig. 2B), in MH-S cells (79.1%, Fig. 2C), and in HeLa cells (91.55%, Fig. 2D). Of interest, in HeLa cells the Δ*stmp1* mutant growth defect started at 2 days postinfection (dpi) and became more pronounced as the infection progressed (Fig. 2D). To measure Δ*stmp1* CCV size, infected cells were stained for LAMP1 by immunofluorescence at 6 dpi and analyzed by microscopy. At 6 dpi, Δ*stmp1* CCVs were significantly smaller (66.9%) than WT CCVs (Fig. 2E and F), suggesting Stmp1 positively influences CCV expansion. Finally, to test whether the observed phenotypes are specific to the lack of Stmp1, the gene was complemented using the endogenous promoter (Fig. S1B). Complementation of the Δ*stmp1* mutant rescued both the growth and CCV phenotype in HeLa cells (Fig. 2D to F) and the growth defect in MH-S cells (data not shown).

10.1128/mBio.03073-21.1 FIG S1 Generation of a Δ*stmp1* mutant bacteria. (A) PCR confirming deletion of *stmp1* (CBU1206) by targeted gene deletion using a loop-in/loop-out strategy. (B) PCR confirming Stmp1 complementation in C. burnetii Δ*stmp1* mutant bacteria using specific primers. Download FIG S1, TIF file, 1.8 MB. Copyright © 2022 Clemente et al. 2022 Clemente et al. https://creativecommons.org/licenses/by/4.0/ This content is distributed under the terms of the Creative Commons Attribution 4.0 International license.

**FIG 3:**
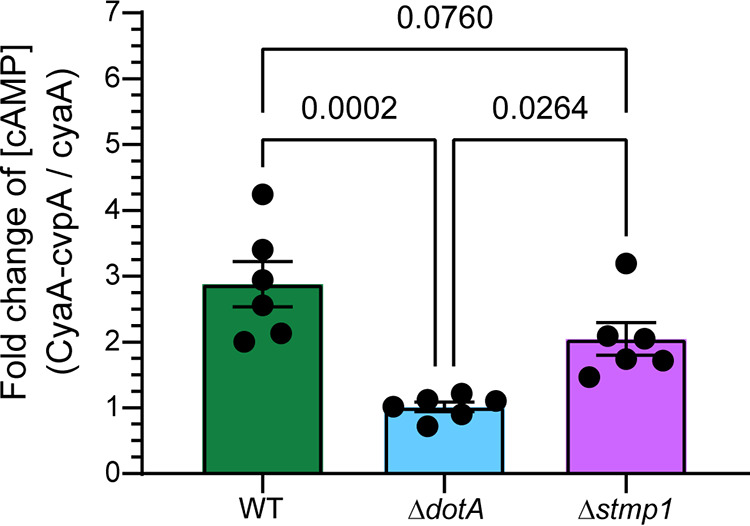
Δ*stmp1* mutant bacteria have a functional T4BSS. Intracellular cAMP levels were determined following infection of HeLa cells with C. burnetii WT, Δ*dotA* (T4BSS mutant), or the Δ*stmp1* mutant expressing CyaA alone or fused to CvpA (T4BSS effector protein). The expression of each C. burnetii transformant was confirmed by Western blotting analysis (data not shown). Results are expressed as the fold change over cAMP levels obtained from infection with C. burnetii expressing CyaA alone. Compared to the C. burnetii Δ*dotA* T4BSS mutant, both C. burnetii WT and Δ*stmp1* mutant bacteria secrete CyaA-CvpA into the host cytoplasm. Data are shown as the mean ± SEM of six independent experiments. Statistical significance was determined by one-way ANOVA with Tukey’s *post hoc* test.

C. burnetii utilizes a specialized Dot/Icm type 4B secretion system (T4BSS) to inject bacterial effector proteins across the CCV membrane and into the host cell cytoplasm in order to manipulate host signaling and trafficking (6, 7, 31). Given the intracellular growth defect of the Δ*stmp1* mutant (Fig. 2B to D), we tested whether Δ*stmp1* mutant bacteria possess a functional T4BSS using an adenylate cyclase (CyaA) translocation assay. HeLa cells were infected with WT, a T4BSS-defective mutant (Δ*dotA*), or Δ*stmp1* mutant bacteria, all expressing either CvpA (a known C. burnetii T4BSS [32]) fused to CyaA, or CyaA alone. Effector translocation was determined by measuring changes in host cAMP levels based on the ratio of CyaA-CvpA/CyaA at 2 dpi (6). As expected, cAMP levels were significantly elevated in WT-infected cells compared to those of Δ*dotA* mutant-infected cells (Fig. 3). The cAMP levels in Δ*stmp1* mutant-infected cells were significantly higher than those of Δ*dotA* mutant-infected cells and similar to those of WT-infected cells, demonstrating that the Δ*stmp1* mutant has a functional T4BSS. Taken together, these data demonstrate that Stmp1 is required for C. burnetii intracellular growth and CCV expansion.

### Stmp1 is critical for CCV fusion with late endosomes and autophagosomes and proper acidification of the CCV.

**FIG 4:**
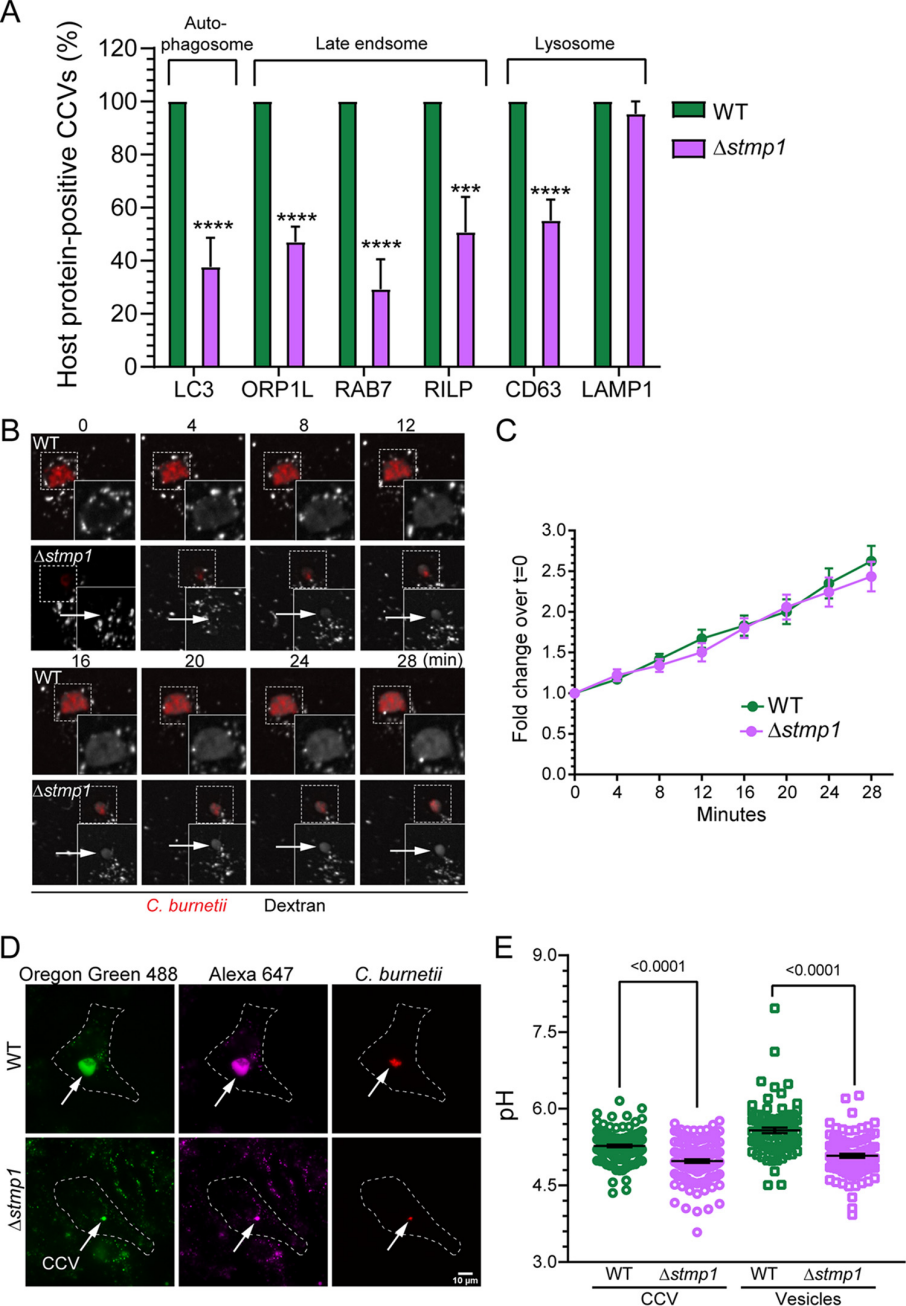
Δ*stmp1* CCVs preferentially fuse with lysosomes and become more acidic. (A) Quantification of CCVs positive for markers of host autophagosomes, late endosomes, and lysosomes indicates that Δ*stmp1* CCVs are deficient in autophagosome and late endosome markers. Infected cells were stained by immunofluorescence for CD63 or LAMP1 or transfected with LC3-GFP, ORP1L-GFP, RAB7-GFP, or RILP-GFP and analyzed by fixed microscopy. Images were visually scored for the presence or absence of the host proteins on the CCV at 3 dpi. Data are shown as the mean ± SEM of at least 20 CCVs in each of three independent experiments. Statistical significance was determined by multiple *t* tests; ***, *P* < 0.005; ****, *P* < 0.001. (B) Representative images of fluorescence dextran trafficking to the CCV at 3 dpi. mCherry-expressing WT- or Δ*stmp1* mutant-infected HeLa cells were pulsed with Alexa 488 dextran for 10 min, followed by live cell spinning disk confocal microscopy, where the cells were imaged at 0 min postpulse and then every 4 min for 28 min. (C) Quantification of changes in dextran fluorescence intensity reveal no significant difference in dextran trafficking to WT and Δ*stmp1* CCVs. The fluorescence intensity of Alexa 488 dextran was measured from an identical region of interest (ROI) within the CCV at each time point. The mean fold change of fluorescence intensity over the initial time point (*t* = 0) was plotted against time. Data are shown as the mean ± SEM of at least 20 CCVs in each of three independent experiments. Statistical significance was determined by multiple *t* tests. (D) The pH of CCV and host cell endosomes was determined at 3 dpi using a ratiometric fluorescence assay. mCherry-expressing WT- or Δ*stmp1* mutant-infected HeLa cells were labeled with Oregon green 488 and Alexa Fluor 647 dextran for 4 h followed by a 1-h chase. (E) Z-stacked images were acquired by live cell spinning disk confocal microscopy, and Oregon green 488, and Alexa 647 intensities were quantitated for each CCV and host cell endosomes and compared to a standard curve to generate individual CCV pH measurements and mean endosomal pH. Δ*stmp1* CCVs are significantly more acidic than WT CCVs. Increased acidification of mature endosomes in Δ*stmp1* mutant-infected cells indicates that Δ*stmp1* mutant bacteria are unable to completely block endosomal maturation at 3 dpi. Data are shown as the mean ± SEM of at least 30 cells in each of three independent experiments. Each circle represents an individual cell or CCV. Statistical significance was determined by one-way ANOVA with Tukey’s *post hoc* test.

CCV biogenesis requires a dynamic T4BSS-dependent process of vesicular trafficking and fusion events. The resulting mature CCV is a large acidic compartment with a hybrid membrane consisting of both bacterial and host components (4, 11). Given the small size of Δ*stmp1* CCVs (Fig. 2E and F), we next assessed whether Δ*stmp1* CCVs acquire known CCV markers on its membrane, which would indicate typical CCV development. Infected cells were stained for CD63 and LAMP1 by immunofluorescence or transfected with GFP-LC3, GFP-ORP1L, GFP-Rab7, or GFP-RILP and then analyzed at 3 dpi by microscopy (4, 5, 33–37). As expected, most WT CCVs were positive for markers of autophagosomes, late endosomes, and lysosomes at 3 dpi (Fig. 4A). However, the autophagosome marker LC3, as well as the late endosome markers ORP1L and Rab7, localized to less than 50% of Δ*stmp1* CCVs, while approximately 65% of Δ*stmp1* CCVs were positive for RILP and CD63, late endosome and late endosome/lysosome markers, respectively. Only the lysosomal marker LAMP1 was detected in 100% of the Δ*stmp1* CCVs (Fig. 4A).

The absence of known CCV markers, as well as their small size (Fig. 2E and F), suggested that Δ*stmp1* CCVs have a fusion defect with the host endocytic pathway. To test this possibility, we measured CCV fusogenicity using a quantitative dextran trafficking assay (38). Dextran is internalized by the cells through fluid-phase endocytosis and delivered to the CCV lumen by fusion between endosomes and the CCV (5). HeLa cells infected with either mCherry-expressing WT or Δ*stmp1* mutant bacteria were pulsed with fluorescent Alexa Fluor 488 dextran for 10 min and then imaged every 4 min for 28 min by live-cell confocal microscopy. Dextran accumulation in the CCV lumen was quantitated by measuring the fold change in fluorescence intensity at every time point over the initial time point. Surprisingly, we found that despite their small size, the Δ*stmp1* CCVs were as fusogenic as WT CCVs (Fig. 4B and C). This enabled us to use our established ratiometric assay to measure the pH of Δ*stmp1* CCVs, as this approach also relies on dextran trafficking to the CCV. HeLa cells infected with either mCherry-expressing WT or Δ*stmp1* mutant bacteria were pulsed for 4 h with pH-sensitive Oregon green 488 dextran and pH-stable Alexa Fluor 647 dextran (39), followed by 1-h chase to allow for endosomal maturation prior to measuring the pH of both CCVs and host endosomes (40) (Fig. 4D). The Δ*stmp1* CCVs (pH ∼4.9) were significantly more acidic than WT CCVs (pH ∼5.2) (Fig. 4E), suggesting that, unlike WT CCVs, Δ*stmp1* CCVs have an acidic pH similar to lysosomes.

We recently demonstrated that the C. burnetii T4BSS blocks host cell endolysosomal maturation, resulting in less acidic mature endosomes and decreased host lysosomes (41). While mature endosomes of WT-infected cells have a pH of ∼5.6, we found that mature endosomes of Δ*stmp1* mutant-infected cells were more acidic (pH ∼5.1), suggesting that the Δ*stmp1* mutant does not efficiently block endosomal maturation (Fig. 4E). To further explore this, we quantitated the endolysosomal content of mock-, C. burnetii WT-, and Δ*dotA* and Δ*stmp1* mutant-infected HeLa cells using the early endosomal marker EEA1 and the lysosomal marker LAMP1. The EEA1 and LAMP1 fluorescence intensities were measured at 3 dpi and normalized to cell area. Confirming our previous results, we found comparable EEA1 levels in all groups and reduced LAMP1 intensity in WT-infected cells compared to both mock- and Δ*dotA* mutant-infected cells (Fig. S2). We observed an intermediate phenotype in Δ*stmp1* mutant-infected cells, with the LAMP1 intensity being significantly higher than that of WT-infected cells but reduced compared to mock- and Δ*dotA* mutant-infected cells. This suggests that the C. burnetii Δ*stmp1* mutant only partially inhibits endolysosomal maturation, leading to an increase in the number of lysosomes available to fuse with the CCV.

10.1128/mBio.03073-21.2 FIG S2 The Δ*stmp1* mutant partially blocks the endolysosomal maturation. (A) Representative images of EEA1 and LAMP1 immunofluorescent staining in mock and infected HeLa cells. (B) Quantitation of EEA1 and LAMP1 intensity at 3 dpi, normalized to cell area. Each circle represents an individual cell. Data are shown as the mean ± SEM of at least 20 cells per condition in each of three independent experiments. Statistical significance was determined by one-way ANOVA with Tukey’s *post hoc* test; ****, *P* < 0.0001. Download FIG S2, TIF file, 2.2 MB. Copyright © 2022 Clemente et al. 2022 Clemente et al. https://creativecommons.org/licenses/by/4.0/ This content is distributed under the terms of the Creative Commons Attribution 4.0 International license.

To further examine whether the lack of Stmp1 affects CCV membrane dynamics, we used live cell imaging to visualize changes in CCV morphology. HeLa cells expressing LAMP1-GFP were infected with either mCherry-expressing WT or Δ*stmp1* mutant bacteria and imaged every 30 min for 24 h by live-cell microscopy. Despite the small size observed for Δ*stmp1* CCVs (Fig. 2E and F), they appear as dynamic as WT CCVs (Video S1). Taken together, these data suggest that Δ*stmp1* CCVs have an altered membrane composition, are dynamic, and preferentially fuse with lysosomes, which enhances their acidification and likely leads to bacterial death.

10.1128/mBio.03073-21.5 Video S1 HeLa cells infected with WT or Δ*stmp1* mutant bacteria. HeLa cells expressing LAMP1-GFP (green) infected with mCherry-expressing WT or Δ*stmp1* mutant bacteria were imaged for 24 h, starting at 3 dpi, using live cell microscopy. Download Movie S1, MOV file, 0.8 MB. Copyright © 2022 Clemente et al. 2022 Clemente et al. https://creativecommons.org/licenses/by/4.0/ This content is distributed under the terms of the Creative Commons Attribution 4.0 International license.

### Stmp1 influences CCV cholesterol levels.

**FIG 5:**
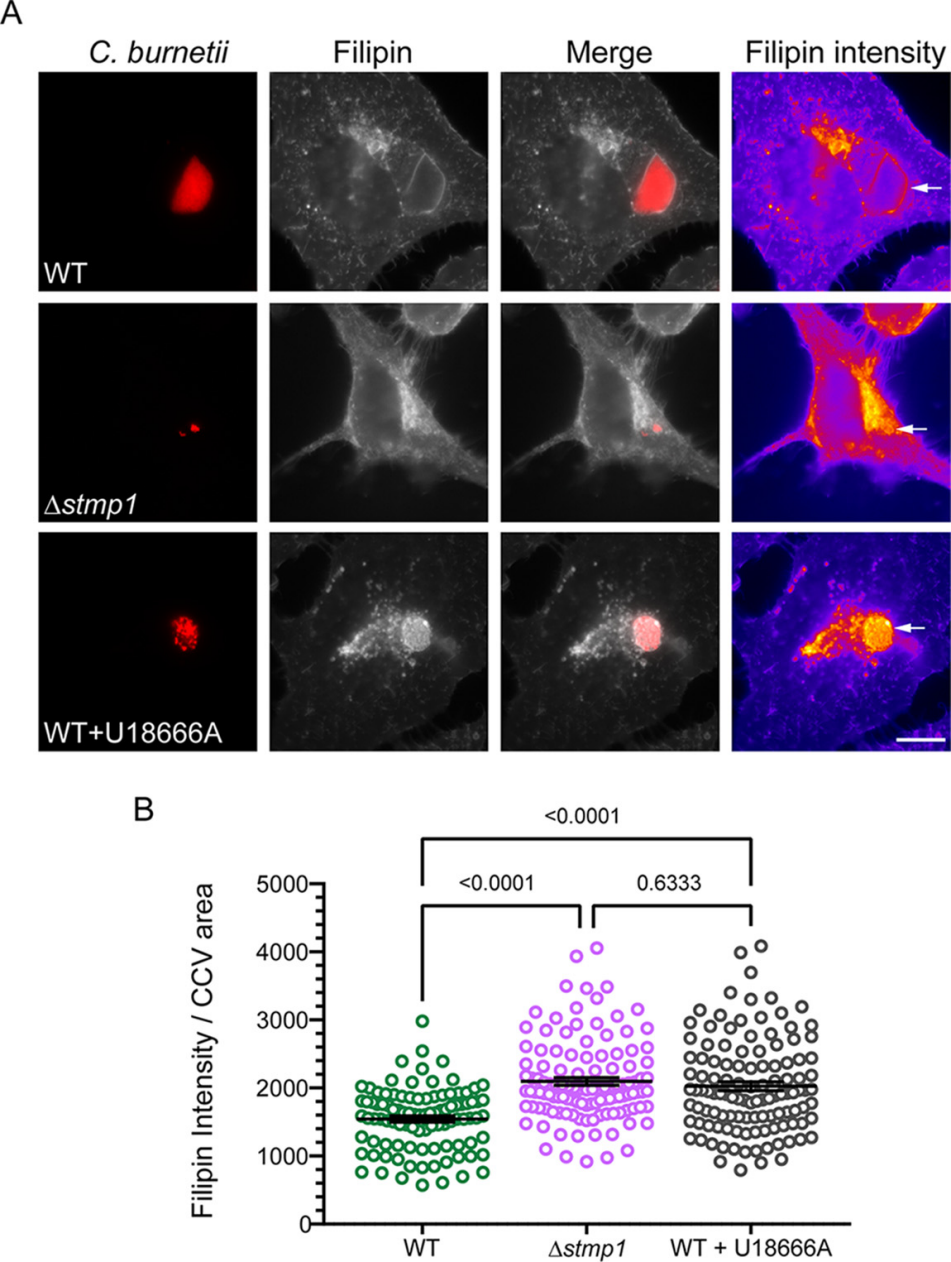
The Δ*stmp1* mutant CCV accumulates cholesterol. Relative cholesterol levels, measured by filipin labeling, indicate that the Δ*stmp1* CCVs have elevated cholesterol. HeLa cells were infected with mCherry-expressing WT or Δ*stmp1* mutant bacteria and sterols stained at 3 dpi using filipin. As a positive control, WT-infected cells were treated for 4 h with 5 μM U18666A, a drug that traps cholesterol in lysosomes and CCVs. (A) Filipin is shown as a heat map, with yellow showing the highest filipin intensity and blue showing the lowest filipin intensity. The white arrows point to the CCVs. Bars = 10 μm. (B) Filipin intensity was measured using ImageJ and normalized to the CCV area. Each circle represents an individual CCV. Data are shown as the mean ± SEM of at least 30 CCVs per condition in each of three independent experiments. Statistical significance was determined by one-way ANOVA with Dunnett’s *post hoc* test.

Given the similarities between the Δ*stmp1* CCV phenotype and our previous studies demonstrating that elevated CCV cholesterol acidifies the CCV (10), we hypothesized that Stmp1 lowers CCV membrane cholesterol levels. To test this, we determined the relative cholesterol levels in WT and Δ*stmp1* CCVs by labeling infected cells with the fluorescent sterol-binding compound filipin. As a positive control, the infected cells were treated with U18666A, a drug that traps cholesterol in lysosomes as well as the CCV membrane (10). CCV filipin labeling was quantitated by measuring the fluorescence intensity from fixed-cell microscopy images. As expected, after U18666A treatment there was a significant increase in filipin intensity in WT CCVs compared to untreated WT-infected cells, indicating cholesterol accumulation (Fig. 5A and B). Filipin levels were significantly higher in Δ*stmp1* CCVs than in WT CCVs (Fig. 5B), indicating that the Δ*stmp1* CCV accumulates cholesterol. Importantly, there is no correlation between the CCV size and cholesterol levels (Fig. S3). Therefore, these findings further support our hypothesis that C. burnetii Stmp1 lowers CCV membrane cholesterol.

10.1128/mBio.03073-21.3 FIG S3 CCV cholesterol levels are not dependent on CCV area. There is no correlation between the filipin intensity and CCV area. Pearson’s correlation is not significant (*P* = 0.8422). Download FIG S3, JPG file, 0.5 MB. Copyright © 2022 Clemente et al. 2022 Clemente et al. https://creativecommons.org/licenses/by/4.0/ This content is distributed under the terms of the Creative Commons Attribution 4.0 International license.

**FIG 6:**
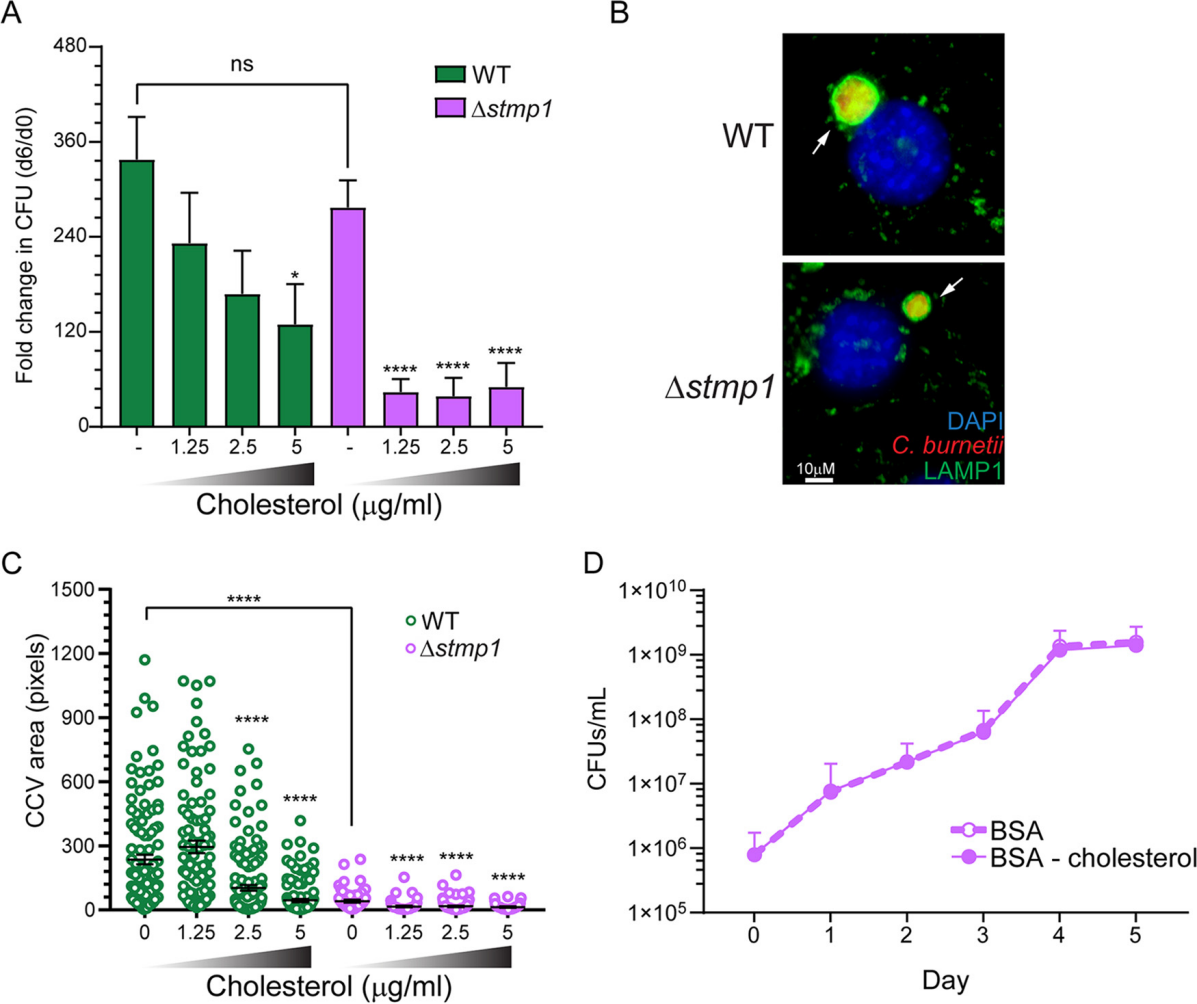
The C. burnetii Δ*stmp1* mutant is hypersensitive to cellular cholesterol. Cholesterol-free DHCR24^−/−^ MEF cells were infected with WT or Δ*stmp1* mutant bacteria for 2 h, followed by treatment with different cholesterol concentrations (0, 1.25, 2.5, or 5 μg/mL). (A) Growth over 6 days indicates that while Δ*stmp1* mutant bacteria grow similarly to WT in the absence of cholesterol, they are sensitive to low cholesterol levels which do not affect WT bacteria. The fold change over day 0 was determined by CFU assay at 6 dpi. Statistical significance was determined by one-way ANOVA test with Dunnett’s *post hoc* test; *, *P* < 0.05; ****, *P* < 0.001. (B) CCVs were stained using immunofluorescence at 6 dpi using LAMP1 (green) and (C) their size was measured using ImageJ. Δ*stmp1* CCVs were smaller than WT CCVs, regardless of cellular cholesterol levels. Each circle represents an individual CCV. Data are shown as the mean ± SEM of at least 30 CCVs per condition in each of three independent experiments. Values that are significantly different from the control value (no cholesterol) were determined by one-way ANOVA; *, *P* < 0.05; ****, *P* < 0.001. (D) Δ*stmp1* mutant bacteria are not sensitive to cholesterol outside the host cell, as demonstrated by growth in axenic cultures. BSA-cholesterol or BSA alone was added to WT or Δ*stmp1* mutant bacteria axenic cultures, and bacterial viability was determined by CFU assay over 5 days. Statistical significance was determined by multiple *t* tests.

Given the cholesterol accumulation on Δ*stmp1* CCVs (Fig. 5A and B), we tested whether host cholesterol levels influence CCV formation and growth of Δ*stmp1* mutant bacteria. Using our established cholesterol-free cell model system based on mouse embryonic fibroblast cells (MEFs) lacking DHCR24, the final enzyme in cholesterol biosynthesis (16), we measured Δ*stmp1* mutant growth and CCV size under different cholesterol concentrations. Interestingly, in cholesterol-free cells, the Δ*stmp1* mutant grew similarly to WT bacteria (Fig. 6A). However, even under low cholesterol concentrations (1.25 to 2.5 μM) that do not affect WT bacteria, growth of the Δ*stmp1* mutant was significantly impaired (Fig. 6A). Furthermore, low cholesterol concentrations (1.25 μM) did not affect the WT CCV size, while the Δ*stmp1* CCVs were significantly smaller than CCVs in cholesterol-free cells (Fig. 6B and C). The CCV size of both WT and Δ*stmp1* mutant bacteria was smaller in DHCR24^−/−^ MEFs treated with higher cholesterol concentrations (2.5 and 5 μM). Surprisingly, while WT and Δ*stmp1* mutant bacteria grew similarly in the absence of cholesterol (Fig. 6A), the average Δ*stmp1* CCV size in cholesterol-free MEFs was at least 5 times smaller than WT CCVs. However, we and others have observed that CCV size and bacterial growth do not always correlate (35, 42, 43). Next, to determine whether cholesterol directly affects the Δ*stmp1* mutant, we assessed axenic growth of Δ*stmp1* mutant bacteria in the presence or absence of cholesterol. Similar to WT C. burnetii (10), cholesterol had no effect on Δ*stmp1* mutant growth in axenic media (Fig. 6D). These data suggest that the poor intracellular growth of the Δ*stmp1* mutant is related to cellular cholesterol levels, and not a direct effect on the bacteria.

### 25-hydroxycholesterol accumulates in Δ*stmp1* mutant-infected cells, blocking C. burnetii growth in a dose-dependent manner.

**FIG 7:**
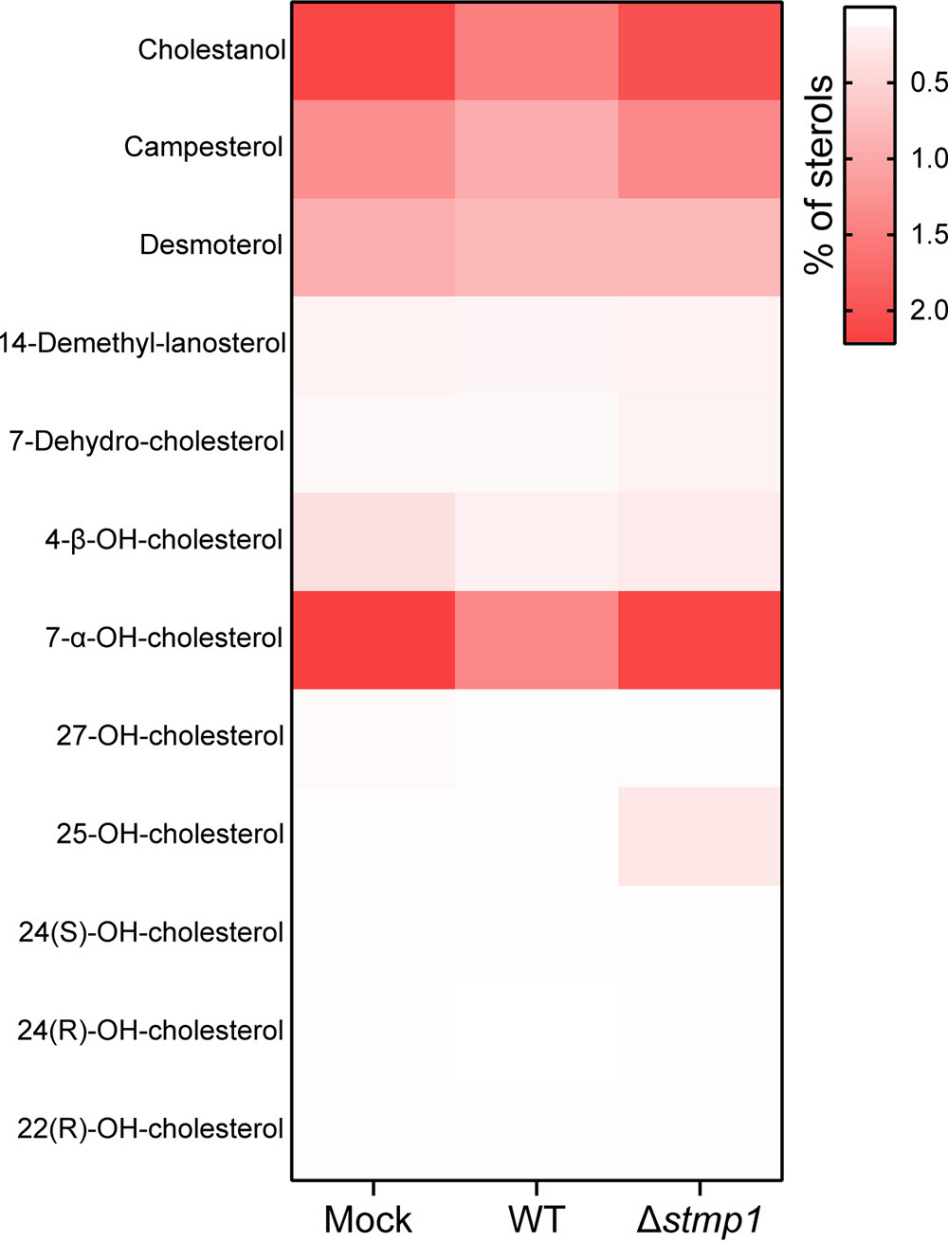
25-HC accumulates in C. burnetii Δ*stmp1* mutant-infected cells. Heat map of the percentage of sterols in mock-, mCherry-expressing WT-, or Δ*stmp1* mutant-infected MH-S cells at 3 dpi. Data are shown as mean values from four independent experiments and normalized to the number of cells. Statistical significance was determined by one-way ANOVA with Dunnett’s *post hoc* test.

**FIG 8:**
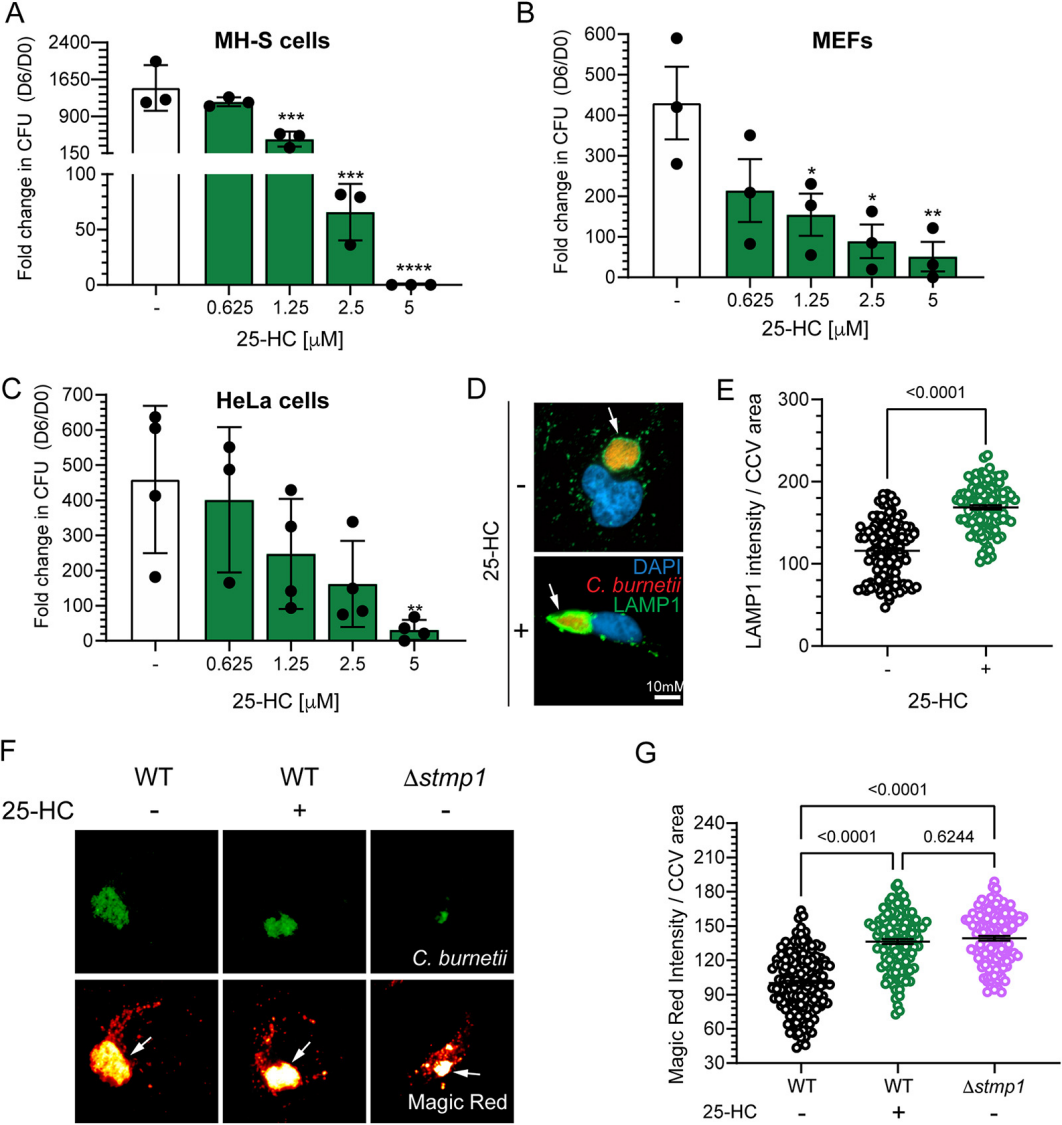
25-HC blocks C. burnetii growth by increasing proteolytic activity in the CCV. (A to C) 25-HC inhibits C. burnetii growth in a concentration-dependent manner in (A) MH-S macrophages, (B) DHCR24^−/−^ MEFs, and (C) HeLa cells. Cells were infected with C. burnetii WT and treated with vehicle control (–) or different 25-HC concentrations (0, 0.625, 1.25, 2.5, or 5 μM). The fold change in growth over day 0 was determined by CFU assay at 6 dpi. Statistical significance was determined by one-way ANOVA test with Dunnett’s *post hoc* test; *, *P* < 0.05; **, *P* < 0.01, ***, *P* < 0.005; ****, *P* < 0.001. (D) Representative immunofluorescence images of C. burnetii CCVs (arrows) stained for LAMP1 at 3 dpi in HeLa cells. (E) Quantitation of CCV LAMP1 intensity, normalized to CCV area, indicates 25-HC CCVs have more LAMP1. Each circle represents an individual CCV. Data are shown as the mean ± SEM of at least 30 CCVs per condition in each of three independent experiments. Statistical significance was determined by *t* test. (F) Representative images of HeLa cells infected with clover-expressing WT or Δ*stmp1* mutant bacteria, treated with vehicle control or 25-HC (5 μM) 24 h before imaging. Infected cells were stained with cathepsin B Magic Red at 3 dpi to visualize proteolytically active lysosomes. Magic Red is shown as a heat map, with white showing the highest intensity and red showing the lowest intensity. The white arrows point to the CCVs. Bars = 10 μm. (G) Quantification of Magic Red shows that Δ*stmp1* CCVs and WT CCVs treated with 25-HC are more proteolytically active than untreated WT CCVs. Magic Red intensity was measured using ImageJ and normalized to CCV area. Each circle represents an individual CCV. Data are shown as the mean ± SEM of at least 30 CCVs per condition in each of three independent experiments. Statistical significance was determined by one-way ANOVA with Dunnett’s *post hoc* test.

In order to test whether Stmp1 alters host cell sterol content, we determined the sterol profile of MH-S macrophages either mock-infected or infected with either WT or Δ*stmp1* mutant bacteria. While the levels of most sterols remained similar among uninfected and infected cells, or between uninfected and Δ*stmp1* mutant-infected cells, we found increased levels of 25-hydroxycholesterol (25-HC) in Δ*stmp1* mutant-infected cells compared to mock- and WT-infected cells (Fig. 7). The oxysterol 25-HC plays a central role in mediating cholesterol homeostasis, as it can promote cholesterol efflux and suppress cholesterol biosynthesis and uptake (44, 45). Therefore, we tested whether 25-HC affects C. burnetii intracellular growth. Compared to untreated cells, C. burnetii growth was inhibited by 25-HC in a dose-dependent manner in MH-S cells (Fig. 8A), cholesterol-free DHCR24^−/−^ MEFs (Fig. 8B), and HeLa cells (Fig. 8C). Treatment with 25-HC also led to increased levels of the lysosomal marker LAMP1 on the CCV membrane (Fig. 8D and E) and negatively affected CCV expansion (Fig. S4). As LAMP1 staining suggested 25-HC increased fusion between CCVs and lysosomes, we measured the proteolytic activity of the lysosomal protease cathepsin B in CCVs, using a fluorescence-based Magic Red assay (37, 41). The Magic red detection substrate is membrane-permeable and uses the photostable red fluorophore cresyl violet, which is linked to two cathepsin B target peptide sequences. Following enzymatic cleavage at one or both sites, the cresyl violet generates red fluorescence, and the fluorescence intensity becomes brighter as the enzymatic activity progresses (46). Using live cell confocal microscopy, we analyzed cathepsin B activity in the CCVs of Δ*stmp1* mutant-infected cells and WT-infected cells, with or without 25-HC treatment. Similar to Δ*stmp1* CCVs, the WT CCVs treated with 25-HC had a higher proteolytic activity compared to untreated WT CCVs (Fig. 8F and G). Together, these data suggest that the absence of Stmp1 leads to dysregulated 25-HC levels, which negatively affects bacterial growth by increasing the CCV fusogenicity with the host lysosomes.

10.1128/mBio.03073-21.4 FIG S4 25-HC treatment affects CCV expansion. HeLa cells were infected with WT for 2 h, followed by treatment with vehicle control or 25-HC (5 μM) daily. At 3 dpi, C. burnetii CCVs were stained by immunofluorescence for LAMP1, and the CCV area was measured using ImageJ, with each circle representing an individual CCV. Data are shown as the mean ± SEM of at least 30 CCVs in each of three independent experiments. Statistical significance was determined by *t* test. Download FIG S4, JPG file, 0.4 MB. Copyright © 2022 Clemente et al. 2022 Clemente et al. https://creativecommons.org/licenses/by/4.0/ This content is distributed under the terms of the Creative Commons Attribution 4.0 International license.

## DISCUSSION

C. burnetii is uniquely sensitive to dysregulated host cell cholesterol levels (10, 20). While cholesterol-rich lipid rafts enhance entry into host cells, cholesterol is not essential for C. burnetii replication (16). In fact, high cholesterol levels are detrimental to C. burnetii during the early stages of CCV biogenesis and growth, as cholesterol accumulation leads to increased acidification of the CCV lumen and bacterial death (10). Further, both genetic mutations (e.g., NPC1, a lysosomal cholesterol transporter) and inhibitors which alter cellular cholesterol homeostasis negatively affect C. burnetii growth, indicating the bacteria are sensitive to cholesterol levels within the intracellular niche (20). Based on these data, we hypothesize that C. burnetii tightly regulates CCV cholesterol levels to create an optimal microenvironment for bacterial growth. Here, we characterized C. burnetii sterol modifying protein 1 (Stmp1) as the first non-T4BSS protein that directly modifies a host cell process. The absence of Stmp1 causes cholesterol accumulation and increased CCV-lysosome fusion and acidification, thus inhibiting C. burnetii intracellular growth. Δ*stmp1* mutant bacteria are hypersensitive to cellular cholesterol yet grow at WT levels in the absence of cholesterol. Notably, we found that C. burnetii Δ*stmp1* mutant-infected cells accumulate the cholesterol metabolite 25-hydroxycholesterol (25-HC). Further, 25-HC treatment of C. burnetii WT-infected cells enhances CCV proteolytic activity and decreases bacterial growth, suggesting that Stmp1 plays a role in reducing both CCV cholesterol and 25-HC cellular levels. Together, these data reveal that Stmp1 regulation of host cholesterol homeostasis is essential for C. burnetii intracellular survival.

The replication defect of the Δ*stmp1* mutant implicates cholesterol in heterotypic fusion of early and late endosomes, autophagosomes, and lysosomes with the CCV (4). The C. burnetii T4BSS directs biogenesis of the CCV, which is required to support bacterial replication (5–7, 47). While we show that the growth and CCV defects of the Δ*stmp1* mutant are not due to a dysfunctional T4BSS, a high percentage of Δ*stmp1* CCVs lack several known CCV markers, including the autophagy protein LC3 (33), the late endosomal markers Rab7 (33), RILP, and ORP1L (35), and the late endosomal/lysosomal marker CD63 (37). This suggests that the heterotypic fusion between the host endocytic pathway and the CCV is altered in bacteria lacking Stmp1. In fact, these data, along with the increased levels of LAMP1 on Δ*stmp1* CCVs and higher Δ*stmp1* CCV acidity, indicate that Δ*stmp1* CCVs primarily fuse with lysosomes. It is unclear if increased CCV-lysosome fusion is due to the altered cholesterol composition of the CCV membrane or a change in the pool of endosomes/lysosomes available to fuse with the CCV. In a previous study, we showed that the C. burnetii T4BSS indirectly regulates CCV pH by inhibiting endolysosomal maturation (41). While the Δ*stmp1* mutant has a functional T4BSS, due to the Δ*stmp1* growth defect, fewer effector proteins are likely secreted at later time points of infection. This would explain our data showing that endolysosomal maturation is not fully blocked in the Δ*stmp1* mutant-infected cells at 3 dpi; this results in a larger pool of mature lysosomes available for heterotypic fusion with the CCV. However, we cannot rule out a defect in fusion between Δ*stmp1* mutant CCVs and host endosomes or autophagosomes. Regardless, our data suggest that the intracellular growth defect of the Δ*stmp1* mutant may be partially caused by increased fusogenicity between the CCV and host lysosomes, resulting in CCV acidification and bacterial degradation.

In the absence of Stmp1, cholesterol accumulates in the CCV membrane early during infection. While cholesterol accumulation in the Salmonella*-*containing vacuole and the Anaplasma phagocytophilum-containing vacuole is associated with increased intracellular bacterial replication (48, 49), elevated cholesterol in the *Coxiella* vacuole is bacteriolytic (10). This bacteriolytic effect is not entirely due to increased CCV-lysosome fusion, as drugs which trap cholesterol in the CCV cause rapid CCV acidification (<30 min) followed by bacterial death (10), and it is unlikely that fusion events would acidify the CCV that quickly. Further, C. burnetii is most sensitive to elevated CCV cholesterol at 1 and 2 dpi, prior to CCV expansion (10). The Δ*stmp1* mutant is hypersensitive to cholesterol supplementation compared to WT bacteria, suggesting that Stmp1 reduces CCV cholesterol to avoid its toxic effect. Importantly, as Stmp1 contains 10 potential transmembrane domains, is not secreted through the T4BSS (26), and localizes to the bacterial outer membrane, C. burnetii may directly modify CCV membrane sterols. C. burnetii is found in close contact with the CCV membrane, as this is a requirement for T4BSS secretion of effector proteins into the cytoplasm. As there is no evidence that C. burnetii utilizes cholesterol as a carbon source or incorporates cholesterol into its cell envelope, the bacteria most likely detoxifies CCV cholesterol during interactions with the CCV membrane.

Based on homology, Stmp1 is predicted to reduce sterol double bonds at carbon 24 as a final step of cholesterol biosynthesis, yet C. burnetii does not synthesize cholesterol from host cell precursors (16). However, Stmp1 is an active enzyme with broad substrate specificity, as it can bind and modify yeast sterols to generate ergosterol, the yeast functional homolog of mammalian cholesterol (25). We hypothesize that the Stmp1 enzymatic modification of cholesterol leads to a sterol species that is no longer labeled with filipin. While quantification of specific sterols in isolated CCVs is currently not feasible, total cell sterol profiling indicated that cellular cholesterol levels do not change among mock, WT, or Δ*stmp1* mutant-infected macrophages. In contrast, the oxysterol 25-HC does accumulate in Δ*stmp1* mutant-infected macrophages, indicating that Stmp1 is directly or indirectly involved in host 25-HC metabolism. 25-HC is synthesized from cholesterol by the addition of a hydroxyl group at position 25 carbon and regulates cholesterol biosynthesis through inhibiting sterol-responsive element binding proteins (SREBPs) (50, 51) and activating cholesterol efflux by liver X receptors (LXRs) (52). 25-HC also plays an important role in mediating the immune response against a variety of pathogens, including highly pathogenic viruses such as human immunodeficiency virus (HIV), Ebola virus, Zika virus, and SARS-Cov2 (53, 54). A recent study found that 25-HC, among other oxysterols, provides innate immunity to Listeria monocytogenes and Shigella flexneri by inhibiting their cell-to-cell spread due to mobilization of cell surface-accessible cholesterol (55). Here, we found that 25-HC blocks C. burnetii growth in different cell types, including in cholesterol-free MEFs that likely lack all oxysterols, as these are cholesterol metabolites. A previous study also showed that 25-HC inhibited C. burnetii replication in Vero cells (21). Interestingly, they observed that 25-HC causes an unusually intense LAMP1 labeling on the CCV membrane (21). In agreement with this report, we found that CCVs have a higher LAMP1 intensity with 25-HC treatment, and the CCVs are significantly smaller. Further, by measuring cathepsin B activity using live cell microscopy, we detected increased proteolytic activity in 25-HC-treated CCVs, suggesting that elevated levels of 25-HC in infected cells either directly or indirectly increases fusion between lysosomes and CCVs. While our data do not demonstrate a direct link between 25-HC, proteolytic activity, and bacterial death, elucidating how 25-HC and CCV cholesterol levels lead to C. burnetii degradation may reveal a novel innate immune response against C. burnetii.

In summary, C. burnetii Stmp1 is a unique sterol-modifying enzyme which plays an essential role in regulating host cholesterol homeostasis. Further, 25-HC was revealed as a potential host defense mechanism against C. burnetii. Interestingly, C. burnetii expresses a second putative sterol-modifying enzyme, CBU1158, which has homology to eukaryotic Δ7 sterol reductases. While it is not yet known if CBU1158 also has enzymatic activity, it may work together with Stmp1 to modify CCV cholesterol. Although the enzymatic mechanism of Stmp1 is not yet known, our work reveals that it may be an attractive drug target and further demonstrates C. burnetii’s unique sensitivity to host cholesterol.

## MATERIALS AND METHODS

### Bacteria and mammalian cells.

C. burnetii Nine Mile phase II (NMII clone 4, RSA 439) Δ*stmp1* mutant bacteria were generated by targeted deletion followed by *sacB* counterselection as previously described (56, 57), and the mutant was complemented by single copy using the *stmp1* gene and associated promoter region (56). The mCherry-expressing Δ*stmp1* mutant was generated by electroporating pJB-CAT-1169-mCherry into the Δ*stmp1* mutant as described previously (58). Clover-expressing WT and Δ*stmp1* mutant bacteria were generated by cloning clover into pJB-CAT-proA plasmid followed by electroporation into the bacteria. Wild type (WT), mCherry-expressing WT (10), Δ*stmp1* mutant, mCherry-expressing Δ*stmp1* mutant, clover-expressing WT, clover-expressing Δ*stmp1* mutant, and Δ*stmp1* complement (Δ*stmp1 comp*) were grown for 4 days in ACCM-2, washed twice with phosphate-buffered saline (PBS), and stored as previously described (10). Human cervical epithelial cells (HeLa, ATCC CCL-2; American Tissue Culture Collection, Manassas, Virginia) and mouse alveolar macrophages (MH-S; ATCC CRL-2019) were maintained in RPMI (Roswell Park Memorial Institute) 1640 medium (Corning, Corning, NY) containing 10% fetal bovine serum (FBS; Atlanta Biologicals, Flowery Branch, GA) and 2 mM l-alanyl-l-glutamine (Glutagro, Corning) at 37°C and 5% CO_2_. To obtain human monocyte-derived macrophages (hMDMs), peripheral blood mononuclear cells were isolated from deidentified buffy coats (Indiana Blood Center, Indianapolis, IN) using Ficoll-Paque (GE Healthcare, Chicago, IL). Monocytes were isolated and differentiated in macrophages as described in reference 59. DHCR24^−/−^ mouse embryonic fibroblasts (MEFs) were cultured in fibroblast medium supplemented with a serum-free growth kit (ATCC) as previously described (16). The multiplicity of infection (MOI) was optimized for each bacterial stock, cell type, and infection condition for a final infection of one internalized bacterium per cell.

### Fractionation of C. burnetii.

Fractionation was performed as previously described in reference 27 with modifications. Briefly, 200 mL of Stmp1-3xFlag-expressing C. burnetii was grown for 7 days in ACCM-D, centrifuged at 16,000 × *g* for 15 min at 4°C, washed in ice-cold PBS, and resuspended in 3 mL ice-cold lysis buffer (50 mM Tris-HCl, pH 7.6, 1 mM ethylenediaminetetraacetic acid [EDTA], complete EDTA-free protease inhibitor cocktail tablet [Sigma-Aldrich, St. Louis, MO], and 10% glycerol). Samples were sonicated on ice using a Whatman GE50 ultrasonic sonicator at 50 W for 10 s, followed by 20 s of rest, until the total sonication time reached 3 min. Samples were then centrifuged at 3,220 × *g* for 15 min at 4°C to remove intact bacterial cells. Then, 100 μL of the supernatant was reserved as the whole-cell lysate sample. The remaining supernatant was centrifuged at 100,000 × *g* at 4°C for 1 h to separate the membrane fraction from the cytoplasmic fraction; 100 μL of the cytoplasmic fraction (supernatant) was reserved. The pellet was resuspended in lysis buffer using a 26-G needle and centrifuged at 100,000 × *g* at 4°C to remove residual cytoplasmic proteins. The pellet was then resuspended in 1 mL ice-cold membrane solubilization buffer (50 mM Tris-HCl, pH 7.6, 200 mM MgCl_2_, 1% Triton X-100) using a 26-G needle and then incubated for 2 h on a rotating mixer at 4°C. Samples volumes were adjusted with solubilization buffer to an equivalent total supernatant volume (cytoplasmic fraction), followed by centrifugation at 100,000 × *g* at 4°C for 1 h to separate the inner and outer membranes. Then, 100 μL of the Triton X-100 soluble fraction (inner membrane) was collected, and the insoluble pellet (outer membrane) was immediately resuspended in 4× Laemmli loading buffer in equivalent volumes to the other fractions. All reserved samples were diluted in 4× Laemmli loading buffer sample buffer and boiled at 95°C for 5 min. Protein lysates were resolved by 10% SDS-PAGE and transferred to nitrocellulose membrane (GE Healthcare). The membranes were then probed separately using mouse anti-flag (Sigma-Aldrich 1:500), rabbit anti-DotH/IcmK (1:2,000), rabbit anti-EF-Ts (1:100), rabbit anti-DotB, and rabbit anti-IcmD (1:200) in 2% bovine serum albumin (BSA) in PBS overnight. After washing in TBS-T (TBS containing 0.05% Tween 20), the membranes were incubated with horseradish peroxidase-conjugated anti-mouse (1:1,000; Thermo Fisher Scientific, Waltham, MA) or anti-rabbit (Thermo Fisher Scientific; 1:1,000) secondary antibodies in 4% nonfat milk in TBS-T and developed using enhanced chemiluminescence (ECL) reagent (SuperSignal West Pico PLUS; Thermo Fisher Scientific).

### C. burnetii growth in cell-free medium.

WT or Δ*stmp1* mutant bacteria were diluted to approximately 10^6^ CFU/mL in ACCM-2 with or without BSA or BSA-cholesterol (10), and 6 mL was transferred into a T25 flask and incubated at 37°C in 2.5% O_2_ and 5% CO_2_. Every 24 h for 5 days, 10 μL was removed from the culture and diluted 1:10 in ACCM-2 and plated in serial dilutions onto 0.25% ACCM-2 agarose plates (30). Each of the three experiments was performed in biological duplicate, and the bacteria were spotted in triplicate.

### C. burnetii intracellular growth by CFU assay.

HeLa cells were plated in a 6-well plate (2 × 10^5^ cells per well) and allowed to adhere overnight. Cells were infected with WT, Δ*stmp1* mutant or Δ*stmp1* complement in 0.5 mL RPMI for 2 h, washed extensively with PBS, and scraped into 2 mL of fresh 10% RPMI. Infected cells were replated in a 24-well plate (2.5 × 10^4^ cells/well for day 2, 10^4^ cells/well for day 4, and 5 × 10^3^ cells per well for day 6). To determine the number of internalized bacteria at day 0, 5 × 10^4^ infected cells were spun and lysed in sterile water for 5 min, diluted in ACCM-2 and spotted onto ACCM-2 agarose plates. For the subsequent time points, the attached cells were lysed in sterile water for 5 min. Monocytes were plated (10^5^ cells per well into a 24-well plate), treated for 7 days with 50 ng/mL human macrophage colony-stimulating factor (M-CSF; Fisher Scientific) to differentiate them into hMDMs, and then infected with WT or Δ*stmp1* mutant bacteria in 0.25 mL 10% RPMI for 2 h, washed extensively with PBS, and incubated in 0.5 mL 10% RPMI. MH-S cells plated in a 6-well plate (2 × 10^5^ cells per well) were infected with WT, Δ*stmp1* mutant or Δ*stmp1* complement bacteria in 0.5 mL 10% RPMI for 2 h, washed extensively with PBS, and incubated in 2 mL 10% RPMI. At days 0 and 6 postinfection, both types of cells were lysed in sterile water for 5 min, and the released bacteria were diluted in ACCM-2 and spotted onto 0.25% ACCM-2 agarose plates (30). The plates were incubated for 7 to 9 days at 37°C in 2.5% O_2_ and 5% CO_2_, and the number of colonies was counted to measure bacterial viability. Each of the three experiments was performed in biological duplicate, and the bacteria were spotted in triplicate.

### Quantification of CCV area.

HeLa cells were plated in a 6-well plate (2 × 10^5^ cells/well) and allowed to adhere overnight. Cells were infected with WT, Δ*stmp1* mutant, or Δ*stmp1* complement bacteria for 2 h, washed extensively with PBS, and scraped into 2 mL of 10% RPMI. Infected cells were replated onto coverslips in a 24-well plate (5 × 10^3^ cells per well). At 6 dpi, cells were fixed with 2.5% paraformaldehyde (PFA) for 15 min, washed in PBS, and blocked/permeabilized in 1% BSA and 0.1% saponin in PBS for 20 min. Coverslips were stained with rabbit anti-LAMP1 (1:1,000; Abcam, Cambridge, UK) along with guinea pig anti-C. burnetii (1:2,500) for 1 h followed by Alexa Fluor secondary antibodies (1:1,000; Invitrogen, Carlsbad, CA) for 1 h. Following washing with PBS, coverslips were mounted with ProLong gold with DAPI and visualized on a Leica inverted DMI6000B microscope using a 63× oil immersion objective. Images were captured and processed identically, and the CCV area was measured using ImageJ software. At least 30 CCVs were measured per condition for each of three independent experiments.

### CyaA translocation assay.

Fc gamma receptor (Fcγ) HeLa cells, kindly provided by Stephanie Shames (Kansas State University), were plated in a 24-well plate (5 × 10^4^ cells/well) and allowed to adhere overnight. WT, Δ*dotA* mutant, or Δ*stmp1* mutant bacteria, harboring pJB-CAT-CyaA-CvpA or vector alone (6), were opsonized with rabbit anti-C. burnetii antibody (1:1,000) for 20 min, at room temperature under rotation. The cells were infected with the opsonized bacteria and incubated in 10% RPMI for 48 h. At 2 dpi, the concentration of cAMP in lysates from infected cells was determined using the cAMP enzyme immunoassay (GE Healthcare) as previously described in reference 6.

### Quantitation of CCV markers.

HeLa cells were plated in a 6-well plate (5 × 10^4^ cells per well) and allowed to adhere overnight. Cells were infected with either mCherry-expressing WT or Δ*stmp1* mutant bacteria in 0.5 mL RPMI for 2 h, washed extensively with PBS, and incubated in 2 ml 10% RPMI. At 2 dpi, infected cells were replated onto coverslips in a 24-well plate (5 × 10^4^ cells per well). At 3 dpi, cells were fixed with 2.5% PFA for 15 min, washed in PBS, and blocked/permeabilized in 1% BSA and 0.1% saponin in PBS for 20 min. Coverslips were stained with rabbit anti-LAMP1 (1:1,000; Abcam) or mouse anti-CD63 (1:1,000; BD Biosciences, Franklin Lakes, NJ) for 1 h, followed by Alexa Fluor 488 secondary antibody (1:1,000; Invitrogen) for 1 h. Following washing with PBS, coverslips were mounted with ProLong gold with 4′, 6′-diamidino-2 phenylindole (DAPI) (Thermo Fisher Scientific). Alternatively, HeLa cells (2 × 10^4^ cells per well of a 24-well plate) were reverse transfected with 0.4 μg of pEGFP plasmids encoding ORP1L, Rab7, RILP, or LC3, using Fugene6 (Promega, Madison, WI) according to the manufacturer’s instructions. Approximately 24 h posttransfection, cells were infected with either mCherry-expressing C. burnetii WT or Δ*stmp1* in 0.25 mL 10% RPMI for 2 h, washed extensively with PBS, and incubated in 10% RPMI. At 3 dpi, the coverslips were fixed with 2.5% PFA for 15 min, and coverslips were mounted using ProLong gold with DAPI. Images were visualized and captured on a Leica inverted DMI6000B microscope using 63× oil immersion objective. The CCVs were visually scored as positive or negative to determine host cell protein recruitment. At least 20 CCVs were scored for each condition of three independent experiments.

### Dextran trafficking.

Dextran trafficking and fusion with CCVs were measured as described previously (38). Briefly, HeLa cells were infected with either mCherry-expressing WT or Δ*stmp1* mutant bacteria in a 6-well plate (5 × 10^4^ cells per well). At 2 dpi, cells were trypsinized, resuspended to 3 × 10^5^ cells/mL, and plated onto ibidi-treated channel *μ*-slide VI^0.4^ (9 × 10^3^ cells per channel; ibidi, Verona, WI). On a Nikon spinning disk confocal microscope (60× oil immersion objective) with an Okolab Bold Line stage top incubator, CCVs were identified and marked using NIS elements (Nikon, Melville, NY) prior to labeling with Alexa Fluor 488 dextran (Thermo Fisher Scientific) for 10 min in 10% RPMI (HeLa cells). The cells were washed with PBS 5 to 6 times and replaced with medium. Z-stacked confocal images were obtained for each CCV every 4 min for 28 min (*t* = 0 through 28; 8 time points). The mean dextran fluorescence intensity of an identical region of interest (ROI) within each CCV was quantified for each time point (ImageJ). The fold change of dextran fluorescence intensity over the initial time point (*t* = 0) was plotted against time. At least 20 CCVs were imaged per condition for each of three independent experiments.

### CCV and vesicular pH measurements.

The CCV pH was measured as described previously (39) with modifications. Briefly, HeLa cells (5 × 10^4^ cells per well) were infected with either mCherry-expressing WT or Δ*stmp1* mutant bacteria in a 6-well plate for 2 h, washed extensively with PBS, and incubated in 10% RPMI. At 2 dpi, cells were trypsinized, resuspended to 3 × 10^5^ cells/mL, and plated onto ibidi-treated channel *μ*-slide VI^0.4^ (9 × 10^3^ cells per channel). The next day cells were labeled with pH-sensitive Oregon green 488 dextran (Thermo Fisher Scientific) and pH-stable Alexa Fluor 647 dextran (Thermo Fisher Scientific) at a final concentration of 0.5 mg/mL in 10% RPMI for 4 h, followed by a 1-h chase to allow for endosomal maturation. After being washed with PBS, cells were incubated in 10% RPMI, and individual CCVs were imaged live using z-stacks of 0.2-μm steps with a Nikon spinning disk confocal microscope (60× oil immersion objective) and an Okolab Bold Line stage-top incubator for environmental control (Okolab, Pozzuoli, Italy). Images were captured and processed identically, fluorescence intensity from maximum-intensity projections was measured for Oregon green 488 and Alexa Fluor 647 using ImageJ (Fiji [60]), and the 488/647 ratio was calculated. For measuring endosomal pH from mock-infected cells, the 488/647 ratio of the entire cell was calculated, whereas for the same analysis from C. burnetii*-*infected cells, the CCV was excluded from the cell area. To generate a pH standard curve, WT C. burnetii-infected HeLa cells were incubated in equilibration buffer (143 mM KCl, 5 mM glucose, 1 mM MgCl_2_, 1 mM CaCl_2_, and 20 mM HEPES) containing the ionophores nigericin (10 μM) and monensin (10 μM) for 5 min followed by incubation in standard buffers of pH ranging from 4.0 to 7.0 containing ionophores for 5 min before imaging. At least 20 CCVs were measured at each pH, and the 488/647 ratio was plotted against the pH of the respective buffer to obtain a sigmoidal standard curve. The experimental samples were then interpolated to the standard curve to determine the pH; a standard curve was generated for each individual experiment. At least 20 CCVs or cells were measured for each of 3 independent experiments.

### Quantitation of early endosomes and lysosomes.

HeLa cells were infected with either mCherry-expressing WT or Δ*stmp1* mutant bacteria in a 6-well plate for 2 h, washed extensively with PBS, and incubated in 10% RPMI. At 2 dpi, the infected cells were replated onto coverslips placed in a 24-well plate (5 × 10^4^ cells per well). The next day, cells were fixed in 2.5% PFA for 15 min and blocked/permeabilized for 20 min in 1% bovine serum albumin (BSA) and 0.1% saponin in PBS. Cells were then incubated with mouse anti-EEA1 (1:500; BD Biosciences) and rabbit anti-LAMP1 (1:1,000; Abcam) for 1 h followed by Alexa Fluor secondary antibodies (1:1,000) for 1 h. Following washing with PBS, coverslips were mounted using ProLong gold with DAPI and visualized on a Leica inverted DMI6000B microscope using a 63× oil immersion objective. Images were captured and processed identically, and the fluorescence intensity of EEA1 and LAMP1 was measured (ImageJ) and normalized to cell area, excluding the CCV from intensity measurements. At least 20 cells were measured per condition for each of three independent experiments.

### Filipin labeling for quantification of sterols.

HeLa cells were plated in a 6-well plate (2 × 10^5^ cells/well) and allowed to adhere overnight. Cells were infected with either mCherry-expressing WT or Δ*stmp1* mutant bacteria for 2 h, washed extensively with PBS, and incubated in growth media. At 2 dpi, the infected cells were replated onto coverslips in a 24-well plate (5 × 10^4^ cells per well). The next day cells were treated with dimethyl sulfoxide (DMSO) control or U18666A at 5 μM for 4 h. After the drug treatment, the cells were fixed with 2.5% PFA on ice for 15 min and incubated with 1:100 filipin (5 mg/mL stock in DMSO; Cayman Chemicals, Ann Arbor, MI) in PBS with 1% BSA for 1 h. Following washing with PBS, coverslips were mounted with ProLong gold and visualized on a Leica inverted DMI6000B microscope with a 63× oil immersion objective. Images were captured under identical capture settings and processed identically using ImageJ (30). At least 30 CCVs were measured per condition for each of the three independent experiments.

### C. burnetii growth and CCV size in DHCR24^−/−^ MEFs.

DHCR24^−/−^ MEFs were plated at 10^5^ cells per well in a 6-well plate and allowed to adhere overnight. MEFs were infected with either WT or Δ*stmp1* mutant bacteria for 1 h in 500 μL fibroblast medium supplemented with a serum-free growth kit, washed with PBS to remove extracellular bacteria, and then gently scraped into 3 mL of medium. For the day 0 sample, 1 mL of infected cells was lysed in sterile water for 5 min, and the released bacteria were diluted in ACCM-2 and plated onto 0.25% ACCM-2 agarose plates, while the remaining cells were replated in a 24-well plate (5 × 10^3^ cells per well) under different cholesterol (Synthechol; Sigma-Aldrich) conditions. The medium was changed daily to ensure constant cholesterol concentrations. At 6 dpi, the cells were either lysed in sterile water for 5 min and the released bacteria diluted in ACCM-2 and spotted onto 0.25% ACCM-2 agarose plates or processed for immunofluorescence in order to measure the CCV size, as described above. Each of the three experiments was performed in biological duplicate, and the bacteria were spotted in triplicate for the CFU assay. At least 30 CCVs were measured for each CCV size experiment.

### Sterol profiling.

MH-S cells (2 × 10^5^ cells per well) were infected with either mCherry-expressing WT or Δ*stmp1* mutant bacteria in a 6-well plate for 2 h, washed extensively with PBS, and incubated in 10% RPMI. At 2 dpi, the cells were gently scraped into 2 mL of fresh 10% RPMI, centrifuged at 280 × *g* for 5 min, and resuspended to 10^7^ cells/mL in 1% RPMI. Using a BD-SORP Aria sorter, infected cells were sorted using mCherry fluorescence. Equal numbers of uninfected cells were counted and plated at 2 × 10^5^ cells per well in a 6-well plate in 2 mL of 10% FBS. The next day cells were scraped, counted, and then centrifuged. The pellets were kept at −80C prior to preparation for sterol profiling analysis. Lipids were extracted using the Bligh and Dyer method and analyzed by liquid chromatography tandem mass spectography (LC/MS/MS) at the Purdue Metabolite Profiling Facility–Bindley Bioscience Center. Quantitative analysis of 13 free sterols was done for 7 different experiments, using mass spectrometry standards available from Avanti Polar Lipids. Samples from four independent experiments were analyzed (Table S1). The sterol quantification was shown as a heat map of each indicated sterol percentage related to the total amount of sterols detected. The percentage of cholesterol and lathosterol together was approximately 91.5% of total sterols. Therefore, these two sterols were removed from the heat map to facilitate data interpretation. Statistical significance was determined by one-way analysis of variance (ANOVA) with Dunnett’s *post hoc* test.

10.1128/mBio.03073-21.6 Table S1 Sterol profiling of mock-, mCherry-expressing WT-, or Δ*stmp1* mutant-infected MH-S cells. Download Table S1, XLSX file, 0.04 MB. Copyright © 2022 Clemente et al. 2022 Clemente et al. https://creativecommons.org/licenses/by/4.0/ This content is distributed under the terms of the Creative Commons Attribution 4.0 International license.

### 25-hydroxycholesterol treatment of C. burnetii*-*infected cells.

MH-S cells, DHCR24^−/−^ MEFs, and HeLa cells infected with WT bacteria were extensively washed with PBS and gently scraped into 3 mL of medium. For the day 0 sample, 1 mL of infected cells was lysed in sterile water for 5 min, and the released bacteria were diluted in ACCM-2 and spotted onto 0.25% ACCM-2 agarose plates, while the remaining cells were replated in a 24-well plate (5 × 10^4^ cells per well) and incubated with different concentrations of 25-hydroxycholesterol (25-HC) or vehicle control (ethanol). 25-HC (Avanti Polar Lipids, Alabaster, AL) was reconstituted in ethanol at a final concentration of 10 mM and stored at −20°C. The medium was changed daily to ensure constant 25-HC concentrations. At 6 dpi, infected cells were lysed in sterile water for 5 min, and the released bacteria were diluted in ACCM-2 and spotted onto 0.25% ACCM-2 agarose plates. C. burnetii*-*infected HeLa cells were also replated onto coverslips placed in a 24-well plate (5 × 10^4^ cells per coverslip), and at 3 dpi they were processed for immunofluorescence as described above. Images were captured on a Leica inverted DMI6000B microscope using a 63× oil immersion objective and processed identically, and the fluorescence intensity of LAMP1 on the CCV area was quantitated (ImageJ). At least 30 cells were measured per condition for each of three independent experiments.

### Magic Red assay for cathepsin B activity.

Active cathepsin B was quantitated using Magic Red as described previously (41), with modifications. Briefly, HeLa cells were plated in a 6-well plate (2 × 10^5^ cells/well) and allowed to adhere overnight. Cells were infected with clover-expressing WT or Δ*stmp1* mutant bacteria for 2 h, washed extensively with PBS, and incubated in 10% RPMI. At 2 dpi, cells were trypsinized, resuspended to 3 × 10^5^ cells/mL, and plated onto an ibidi-treated channel *μ*-slide VI^0.4^ (9 × 10^3^ cells per channel). Cells were treated with vehicle control or 25-HC at 5 μM for 24 h. The next day cells were labeled with 50 μL diluted Magic Red (ImmunoChemistry Technologies, Bloomington, MN) in phenol-red free 10% RPMI and incubated for 30 min at 37°C and 5% CO_2_, and individual CCVs were live imaged, with identical capture settings, using z-stacks of 0.3-μm steps with a Nikon spinning disk confocal microscope. The Magic Red fluorescence intensity was measured and normalized to the CCV area. At least 20 CCVs were measured per condition in each of three independent experiments.

### Data analyses.

Image processing and analyses were done in ImageJ (Fiji) software. Statistical analyses were performed using an unpaired Student’s *t* test, ordinary one-way ANOVA (with Tukey’s correction), or multiple *t* tests as appropriate in Prism (GraphPad, La Jolla, CA).

## References

[1] Maurin M, Raoult D. 1999. Q fever. Clin Microbiol Rev 12:518–553. doi:10.1128/CMR.12.4.518. 10515901 PMC88923

[2] Mazokopakis EE, Karefilakis CM, Starakis IK. 2010. Q fever endocarditis. Infect Disord Drug Targets 10:27–31. doi:10.2174/187152610790410918. 20218950

[3] Ackland JR, Worswick DA, Marmion BP. 1994. Vaccine prophylaxis of Q fever. A follow-up study of the efficacy of Q-Vax (CSL) 1985–1990. Med J Aust 160:704–708. doi:10.5694/j.1326-5377.1994.tb125909.x. 8202006

[4] Voth DE, Heinzen RA. 2007. Lounging in a lysosome: the intracellular lifestyle of Coxiella burnetii. Cell Microbiol 9:829–840. doi:10.1111/j.1462-5822.2007.00901.x. 17381428

[5] Heinzen RA, Scidmore MA, Rockey DD, Hackstadt T. 1996. Differential interaction with endocytic and exocytic pathways distinguish parasitophorous vacuoles of Coxiella burnetii and Chlamydia trachomatis. Infect Immun 64:796–809. doi:10.1128/iai.64.3.796-809.1996. 8641784 PMC173840

[6] Beare PA, Gilk SD, Larson CL, Hill J, Stead CM, Omsland A, Cockrell DC, Howe D, Voth DE, Heinzen RA. 2011. Dot/Icm type IVB secretion system requirements for Coxiella burnetii growth in human macrophages. mBio 2:e00175-11. doi:10.1128/mBio.00175-11. 21862628 PMC3163939

[7] Carey KL, Newton HJ, Lührmann A, Roy CR. 2011. The Coxiella burnetii Dot/Icm system delivers a unique repertoire of type IV effectors into host cells and is required for intracellular replication. PLoS Pathog 7:e1002056. doi:10.1371/journal.ppat.1002056. 21637816 PMC3102713

[8] Voth DE, Beare PA, Howe D, Sharma UM, Samoilis G, Cockrell DC, Omsland A, Heinzen RA. 2011. The Coxiella burnetii cryptic plasmid is enriched in genes encoding type IV secretion system substrates. J Bacteriol 193:1493–1503. doi:10.1128/JB.01359-10. 21216993 PMC3067651

[9] Heinzen RA, Hackstadt T, Samuel JE. 1999. Developmental biology of Coxiella burnetii. Trends Microbiol 7:149–154. doi:10.1016/S0966-842X(99)01475-4. 10217829

[10] Mulye M, Samanta D, Winfree S, Heinzen RA, Gilk SD. 2017. Elevated cholesterol in the Coxiella burnetii intracellular niche is bacteriolytic. mBio 8:e02313-16. doi:10.1128/mBio.02313-16. 28246364 PMC5347348

[11] van Schaik EJ, Chen C, Mertens K, Weber MM, Samuel JE. 2013. Molecular pathogenesis of the obligate intracellular bacterium Coxiella burnetii. Nat Rev Microbiol 11:561–573. doi:10.1038/nrmicro3049. 23797173 PMC4134018

[12] Mouritsen OG, Zuckermann MJ. 2004. What’s so special about cholesterol? Lipids 39:1101–1113. doi:10.1007/s11745-004-1336-x. 15726825

[13] Samanta D, Mulye M, Clemente TM, Justis AV, Gilk SD. 2017. Manipulation of host cholesterol by obligate intracellular bacteria. Front Cell Infect Microbiol 7:165–165. doi:10.3389/fcimb.2017.00165. 28529926 PMC5418226

[14] Toledo A, Benach JL. 2015. Hijacking and use of host lipids by intracellular pathogens. Microbiol Spectr 3:VMBF-0001-2014. doi:10.1128/microbiolspec.VMBF-0001-2014. PMC5790186 27337282

[15] Capo C, Lindberg FP, Meconi S, Zaffran Y, Tardei G, Brown EJ, Raoult D, Mege JL. 1999. Subversion of monocyte functions by Coxiella burnetii: impairment of the cross-talk between alphavbeta3 integrin and CR3. J Immunol 163:6078–6085. 10570297

[16] Gilk SD, Cockrell DC, Luterbach C, Hansen B, Knodler LA, Ibarra JA, Steele-Mortimer O, Heinzen RA. 2013. Bacterial colonization of host cells in the absence of cholesterol. PLoS Pathog 9:e1003107. doi:10.1371/journal.ppat.1003107. 23358892 PMC3554619

[17] Mahapatra S, Ayoubi P, Shaw EI. 2010. Coxiella burnetii Nine Mile II proteins modulate gene expression of monocytic host cells during infection. BMC Microbiol 10:244. doi:10.1186/1471-2180-10-244. 20854687 PMC2954873

[18] Ren Q, Robertson SJ, Howe D, Barrows LF, Heinzen RA. 2003. Comparative DNA microarray analysis of host cell transcriptional responses to infection by Coxiella burnetii or Chlamydia trachomatis. Ann N Y Acad Sci 990:701–713. doi:10.1111/j.1749-6632.2003.tb07447.x. 12860710

[19] Mulye M, Zapata B, Gilk SD. 2018. Altering lipid droplet homeostasis affects Coxiella burnetii intracellular growth. PLoS One 13:e0192215. doi:10.1371/journal.pone.0192215. 29390006 PMC5794150

[20] Czyż DM, Potluri L-P, Jain-Gupta N, Riley SP, Martinez JJ, Steck TL, Crosson S, Shuman HA, Gabay JE. 2014. Host-directed antimicrobial drugs with broad-spectrum efficacy against intracellular bacterial pathogens. mBio 5:e01534-14–e01514. doi:10.1128/mBio.01534-14. 25073644 PMC4128363

[21] Howe D, Heinzen RA. 2006. Coxiella burnetii inhabits a cholesterol-rich vacuole and influences cellular cholesterol metabolism. Cell Microbiol 8:496–507. doi:10.1111/j.1462-5822.2005.00641.x. 16469060

[22] Beare PA, Unsworth N, Andoh M, Voth DE, Omsland A, Gilk SD, Williams KP, Sobral BW, Kupko JJ, Porcella SF, Samuel JE, Heinzen RA. 2009. Comparative genomics reveal extensive transposon-mediated genomic plasticity and diversity among potential effector proteins within the genus Coxiella. Infect Immun 77:642–656. doi:10.1128/IAI.01141-08. 19047403 PMC2632050

[23] Frohlich KM, Roberts RAW, Housley NA, Audia JP. 2010. Rickettsia prowazekii uses an sn-glycerol-3-phosphate dehydrogenase and a novel dihydroxyacetone phosphate transport system to supply triose phosphate for phospholipid biosynthesis. J Bacteriol 192:4281–4288. doi:10.1128/JB.00443-10. 20581209 PMC2937374

[24] Moliner C, Raoult D, Fournier PE. 2009. Evidence that the intra-amoebal Legionella drancourtii acquired a sterol reductase gene from eukaryotes. BMC Res Notes 2:51. doi:10.1186/1756-0500-2-51. 19327142 PMC2667531

[25] Gilk SD, Beare PA, Heinzen RA. 2010. Coxiella burnetii expresses a functional Delta24 sterol reductase. J Bacteriol 192:6154–6159. doi:10.1128/JB.00818-10. 20870767 PMC2981196

[26] Burette M, Allombert J, Lambou K, Maarifi G, Nisole S, Di Russo Case E, Blanchet FP, Hassen-Khodja C, Cabantous S, Samuel J, Martinez E, Bonazzi M. 2020. Modulation of innate immune signaling by a Coxiella burnetii eukaryotic-like effector protein. Proc Natl Acad Sci USA 117:13708–13718. doi:10.1073/pnas.1914892117. 32482853 PMC7306807

[27] Kuba M, Neha N, Newton P, Lee YW, Bennett-Wood V, Hachani A, De Souza DP, Nijagal B, Dayalan S, Tull D, McConville MJ, Sansom FM, Newton HJ. 2020. EirA is a novel protein essential for intracellular replication of Coxiella burnetii. Infect Immun 88:e00913-19. doi:10.1128/IAI.00913-19. 32205404 PMC7240097

[28] Vincent CD, Friedman JR, Jeong KC, Buford EC, Miller JL, Vogel JP. 2006. Identification of the core transmembrane complex of the Legionella Dot/Icm type IV secretion system. Mol Microbiol 62:1278–1291. doi:10.1111/j.1365-2958.2006.05446.x. 17040490

[29] Stead CM, Omsland A, Beare PA, Sandoz KM, Heinzen RA. 2013. Sec-mediated secretion by Coxiella burnetii. BMC Microbiol 13:222. doi:10.1186/1471-2180-13-222. 24093460 PMC3882888

[30] Vallejo Esquerra E, Yang H, Sanchez SE, Omsland A. 2017. Physicochemical and nutritional requirements for axenic replication suggest physiological basis for Coxiella burnetii niche restriction. Front Cell Infect Microbiol 7:190. doi:10.3389/fcimb.2017.00190. 28620582 PMC5449765

[31] Seshadri R, Paulsen IT, Eisen JA, Read TD, Nelson KE, Nelson WC, Ward NL, Tettelin H, Davidsen TM, Beanan MJ, Deboy RT, Daugherty SC, Brinkac LM, Madupu R, Dodson RJ, Khouri HM, Lee KH, Carty HA, Scanlan D, Heinzen RA, Thompson HA, Samuel JE, Fraser CM, Heidelberg JF. 2003. Complete genome sequence of the Q-fever pathogen Coxiella burnetii. Proc Natl Acad Sci USA 100:5455–5460. doi:10.1073/pnas.0931379100. 12704232 PMC154366

[32] Larson CL, Beare PA, Howe D, Heinzen RA. 2013. Coxiella burnetii effector protein subverts clathrin-mediated vesicular trafficking for pathogen vacuole biogenesis. Proc Natl Acad Sci USA 110:E4770-9. doi:10.1073/pnas.1309195110. 24248335 PMC3856779

[33] Beron W, Gutierrez MG, Rabinovitch M, Colombo MI. 2002. Coxiella burnetii localizes in a Rab7-labeled compartment with autophagic characteristics. Infect Immun 70:5816–5821. doi:10.1128/IAI.70.10.5816-5821.2002. 12228312 PMC128334

[34] Romano PS, Gutierrez MG, Berón W, Rabinovitch M, Colombo MI. 2007. The autophagic pathway is actively modulated by phase II Coxiella burnetii to efficiently replicate in the host cell. Cell Microbiol 9:891–909. doi:10.1111/j.1462-5822.2006.00838.x. 17087732

[35] Justis AV, Hansen B, Beare PA, King KB, Heinzen RA, Gilk SD. 2017. Interactions between the Coxiella burnetii parasitophorous vacuole and the endoplasmic reticulum involve the host protein ORP1L. Cell Microbiol 19:10.1111/cmi.12637. doi:10.1111/cmi.12637. PMC5177503 27345457

[36] Ortiz Flores RM, Distel JS, Aguilera MO, Berón W. 2019. The role of microtubules and the dynein/dynactin motor complex of host cells in the biogenesis of the Coxiella burnetii-containing vacuole. PLoS One 14:e0209820. doi:10.1371/journal.pone.0209820. 30640917 PMC6331085

[37] Howe D, Shannon JG, Winfree S, Dorward DW, Heinzen RA. 2010. Coxiella burnetii phase I and II variants replicate with similar kinetics in degradative phagolysosome-like compartments of human macrophages. Infect Immun 78:3465–3474. doi:10.1128/IAI.00406-10. 20515926 PMC2916283

[38] Winfree S, Gilk SD. 2017. Quantitative dextran trafficking to the Coxiella burnetii parasitophorous vacuole. Curr Protoc Microbiol 46:6c.2.1–6c.2.12. doi:10.1002/cpmc.34. PMC5558434 28800156

[39] Samanta D, Gilk SD. 2017. Measuring pH of the Coxiella burnetii parasitophorous vacuole. Curr Protoc Microbiol 47:6C 3 1–6C 3 11. doi:10.1002/cpmc.38. PMC9893848 29120485

[40] Huotari J, Helenius A. 2011. Endosome maturation. EMBO J 30:3481–3500. doi:10.1038/emboj.2011.286. 21878991 PMC3181477

[41] Samanta D, Clemente TM, Schuler BE, Gilk SD. 2019. Coxiella burnetii type 4B secretion system-dependent manipulation of endolysosomal maturation is required for bacterial growth. PLoS Pathog 15:e1007855. doi:10.1371/journal.ppat.1007855. 31869379 PMC6953889

[42] Newton HJ, Kohler LJ, McDonough JA, Temoche-Diaz M, Crabill E, Hartland EL, Roy CR. 2014. A screen of Coxiella burnetii mutants reveals important roles for Dot/Icm effectors and host autophagy in vacuole biogenesis. PLoS Pathog 10:e1004286. doi:10.1371/journal.ppat.1004286. 25080348 PMC4117601

[43] Martinez E, Allombert J, Cantet F, Lakhani A, Yandrapalli N, Neyret A, Norville IH, Favard C, Muriaux D, Bonazzi M. 2016. Coxiella burnetii effector CvpB modulates phosphoinositide metabolism for optimal vacuole development. Proc Natl Acad Sci USA 113:E3260–E3269. doi:10.1073/pnas.1522811113. 27226300 PMC4988616

[44] Goldstein JL, DeBose-Boyd RA, Brown MS. 2006. Protein sensors for membrane sterols. Cell 124:35–46. doi:10.1016/j.cell.2005.12.022. 16413480

[45] Waltl S, Patankar JV, Fauler G, Nusshold C, Ullen A, Eibinger G, Wintersperger A, Kratky D, Malle E, Sattler W. 2013. 25-Hydroxycholesterol regulates cholesterol homeostasis in the murine CATH.a neuronal cell line. Neurosci Lett 539:16–21. doi:10.1016/j.neulet.2013.01.014. 23347841 PMC3610018

[46] Van Noorden CJ, Boonacker E, Bissell ER, Meijer AJ, van Marle J, Smith RE. 1997. Ala-Pro-cresyl violet, a synthetic fluorogenic substrate for the analysis of kinetic parameters of dipeptidyl peptidase IV (CD26) in individual living rat hepatocytes. Anal Biochem 252:71–77. doi:10.1006/abio.1997.2312. 9324943

[47] Howe D, Melnicáková J, Barák I, Heinzen RA. 2003. Maturation of the Coxiella burnetii parasitophorous vacuole requires bacterial protein synthesis but not replication. Cell Microbiol 5:469–480. doi:10.1046/j.1462-5822.2003.00293.x. 12814437

[48] Catron DM, Sylvester MD, Lange Y, Kadekoppala M, Jones BD, Monack DM, Falkow S, Haldar K. 2002. The Salmonella-containing vacuole is a major site of intracellular cholesterol accumulation and recruits the GPI-anchored protein CD55. Cell Microbiol 4:315–328. doi:10.1046/j.1462-5822.2002.00198.x. 12067317

[49] Xiong Q, Lin M, Rikihisa Y. 2009. Cholesterol-dependent anaplasma phagocytophilum exploits the low-density lipoprotein uptake pathway. PLoS Pathog 5:e1000329. doi:10.1371/journal.ppat.1000329. 19283084 PMC2654415

[50] Lund EG, Kerr TA, Sakai J, Li WP, Russell DW. 1998. cDNA cloning of mouse and human cholesterol 25-hydroxylases, polytopic membrane proteins that synthesize a potent oxysterol regulator of lipid metabolism. J Biol Chem 273:34316–34327. doi:10.1074/jbc.273.51.34316. 9852097

[51] Adams CM, Reitz J, De Brabander JK, Feramisco JD, Li L, Brown MS, Goldstein JL. 2004. Cholesterol and 25-hydroxycholesterol inhibit activation of SREBPs by different mechanisms, both involving SCAP and Insigs. J Biol Chem 279:52772–52780. doi:10.1074/jbc.M410302200. 15452130

[52] Liu Y, Wei Z, Zhang Y, Ma X, Chen Y, Yu M, Ma C, Li X, Cao Y, Liu J, Han J, Yang X, Duan Y. 2018. Activation of liver X receptor plays a central role in antiviral actions of 25-hydroxycholesterol. J Lipid Res 59:2287–2296. doi:10.1194/jlr.M084558. 30309895 PMC6277154

[53] Liu S-Y, Aliyari R, Chikere K, Li G, Marsden MD, Smith JK, Pernet O, Guo H, Nusbaum R, Zack JA, Freiberg AN, Su L, Lee B, Cheng G. 2013. Interferon-inducible cholesterol-25-hydroxylase broadly inhibits viral entry by production of 25-hydroxycholesterol. Immunity 38:92–105. doi:10.1016/j.immuni.2012.11.005. 23273844 PMC3698975

[54] Zu S, Deng Y-Q, Zhou C, Li J, Li L, Chen Q, Li X-F, Zhao H, Gold S, He J, Li X, Zhang C, Yang H, Cheng G, Qin C-F. 2020. 25-Hydroxycholesterol is a potent SARS-CoV-2 inhibitor. Cell Res 30:1043–1045. doi:10.1038/s41422-020-00398-1. 32811977 PMC7431750

[55] Abrams ME, Johnson KA, Perelman SS, Zhang L-S, Endapally S, Mar KB, Thompson BM, McDonald JG, Schoggins JW, Radhakrishnan A, Alto NM. 2020. Oxysterols provide innate immunity to bacterial infection by mobilizing cell surface accessible cholesterol. Nat Microbiol 5:929–942. doi:10.1038/s41564-020-0701-5. 32284563 PMC7442315

[56] Beare PA, Larson CL, Gilk SD, Heinzen RA. 2012. Two systems for targeted gene deletion in Coxiella burnetii. Appl Environ Microbiol 78:4580–4589. doi:10.1128/AEM.00881-12. 22522687 PMC3370473

[57] Beare PA. 2012. Genetic manipulation of Coxiella burnetii. Adv Exp Med Biol 984:249–271. doi:10.1007/978-94-007-4315-1_13. 22711636

[58] Omsland A, Beare PA, Hill J, Cockrell DC, Howe D, Hansen B, Samuel JE, Heinzen RA. 2011. Isolation from animal tissue and genetic transformation of Coxiella burnetii are facilitated by an improved axenic growth medium. Appl Environ Microbiol 77:3720–3725. doi:10.1128/AEM.02826-10. 21478315 PMC3127619

[59] Clemente TM, Mulye M, Justis AV, Nallandhighal S, Tran TM, Gilk SD. 2018. Coxiella burnetii blocks intracellular interleukin-17 signaling in macrophages. Infect Immun 86:e00532-18. doi:10.1128/IAI.00532-18. 30061378 PMC6204741

[60] Schindelin J, Arganda-Carreras I, Frise E, Kaynig V, Longair M, Pietzsch T, Preibisch S, Rueden C, Saalfeld S, Schmid B, Tinevez J-Y, White DJ, Hartenstein V, Eliceiri K, Tomancak P, Cardona A. 2012. Fiji: an open-source platform for biological-image analysis. Nat Methods 9:676–682. doi:10.1038/nmeth.2019. 22743772 PMC3855844

