# Supplementary figures and images for: Coxiella burnetii Sterol-Modifying Protein Stmp1 Regulates Cholesterol in the Intracellular Niche
Source: mBio. 2022 Jan 25;13(1):e03073-21. doi: 10.1128/mbio.03073-21 (PMC8787468; doi:10.1128/mbio.03073-21)

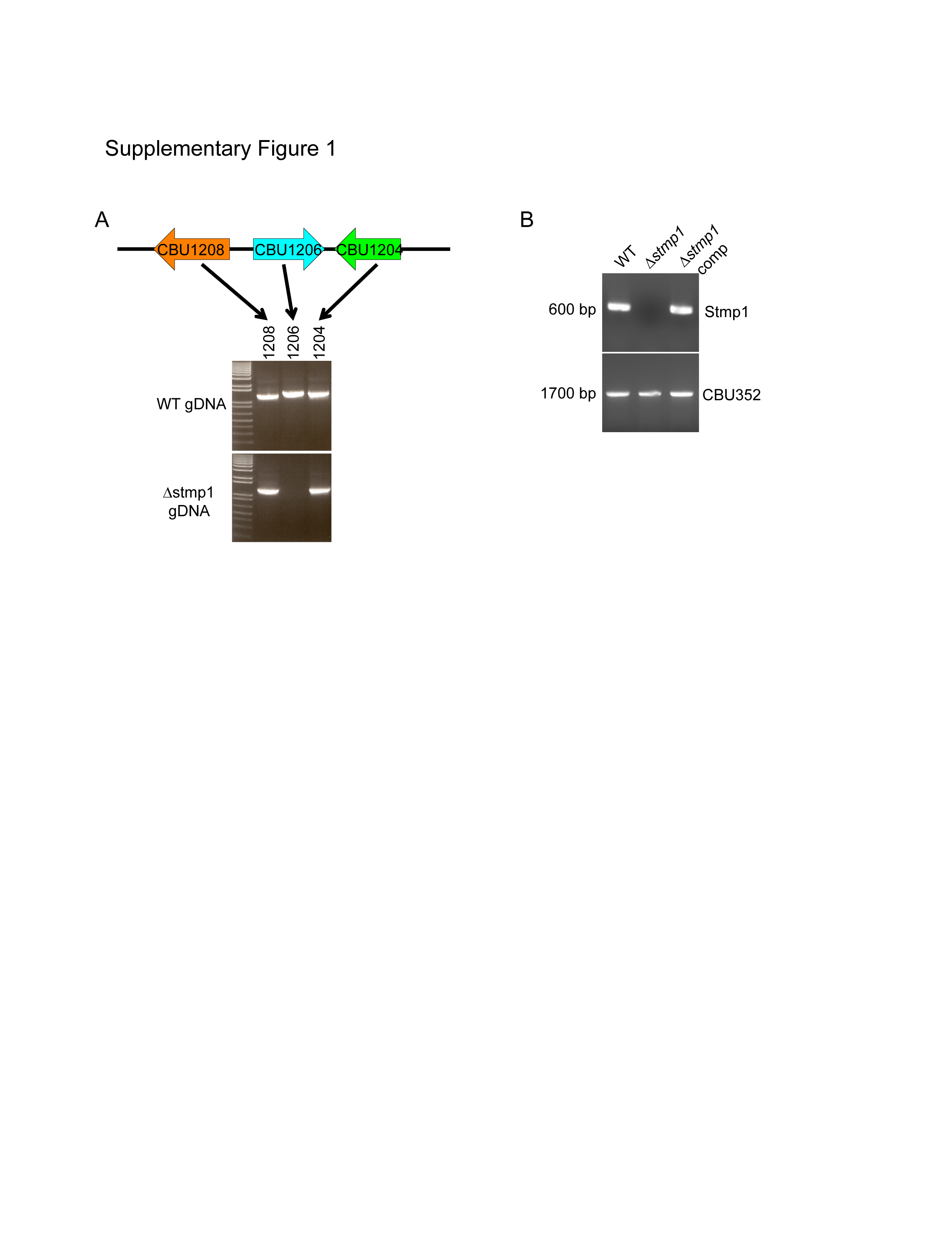

Supplement: FIG S1 [file mbio.03073-21-sf001.tif]

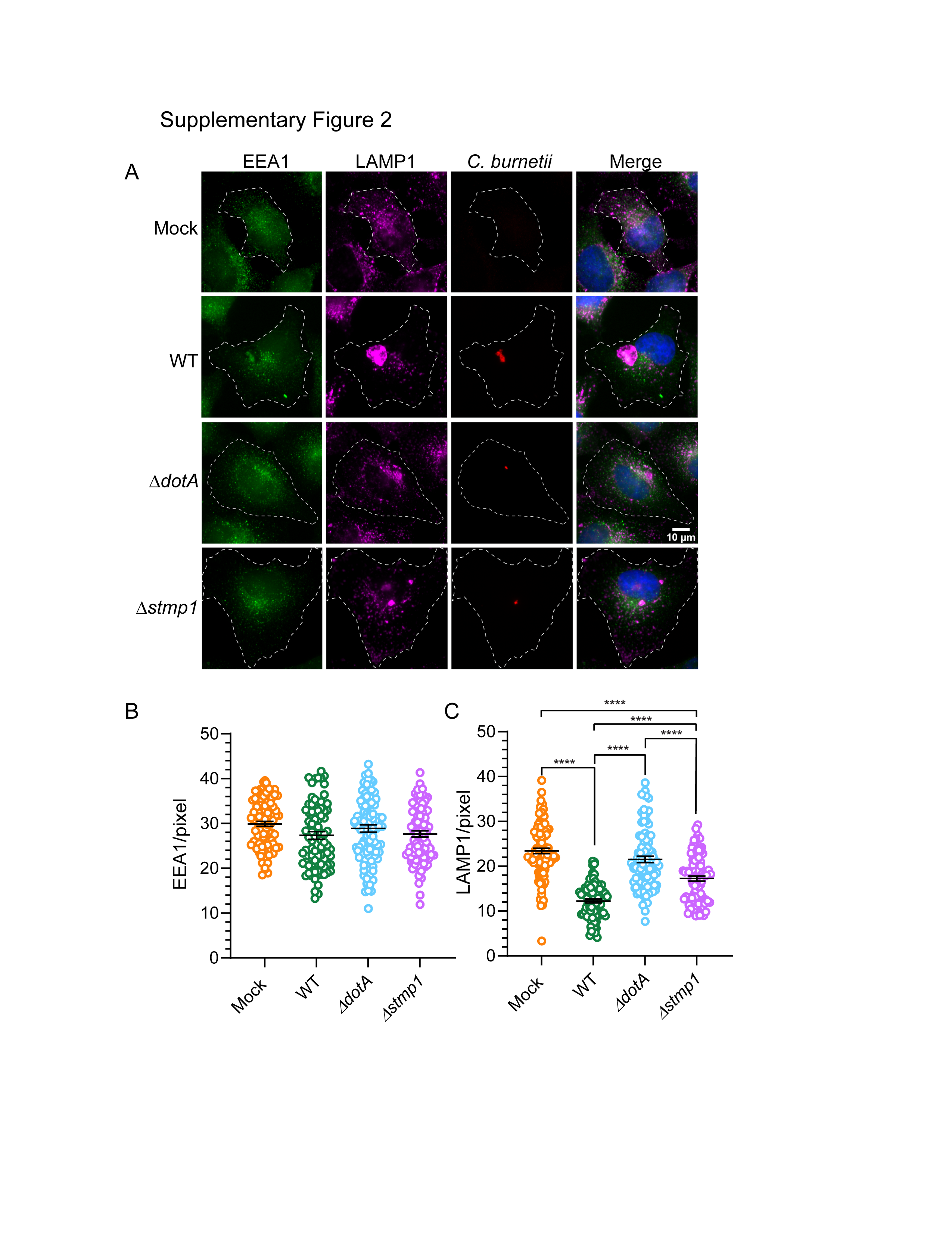

Supplement: FIG S2 [file mbio.03073-21-sf002.tif]

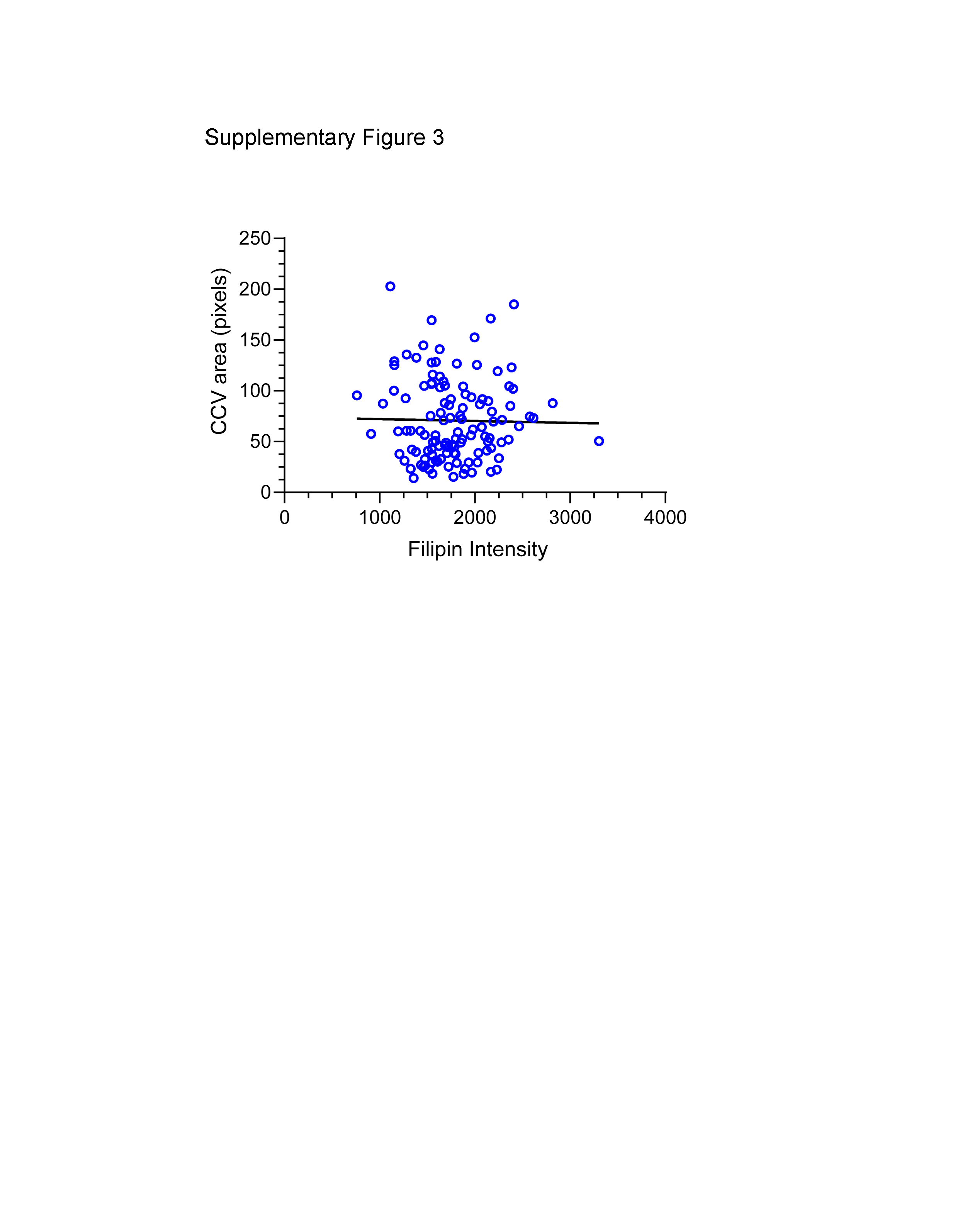

Supplement: FIG S3 [file mbio.03073-21-sf003.jpg]

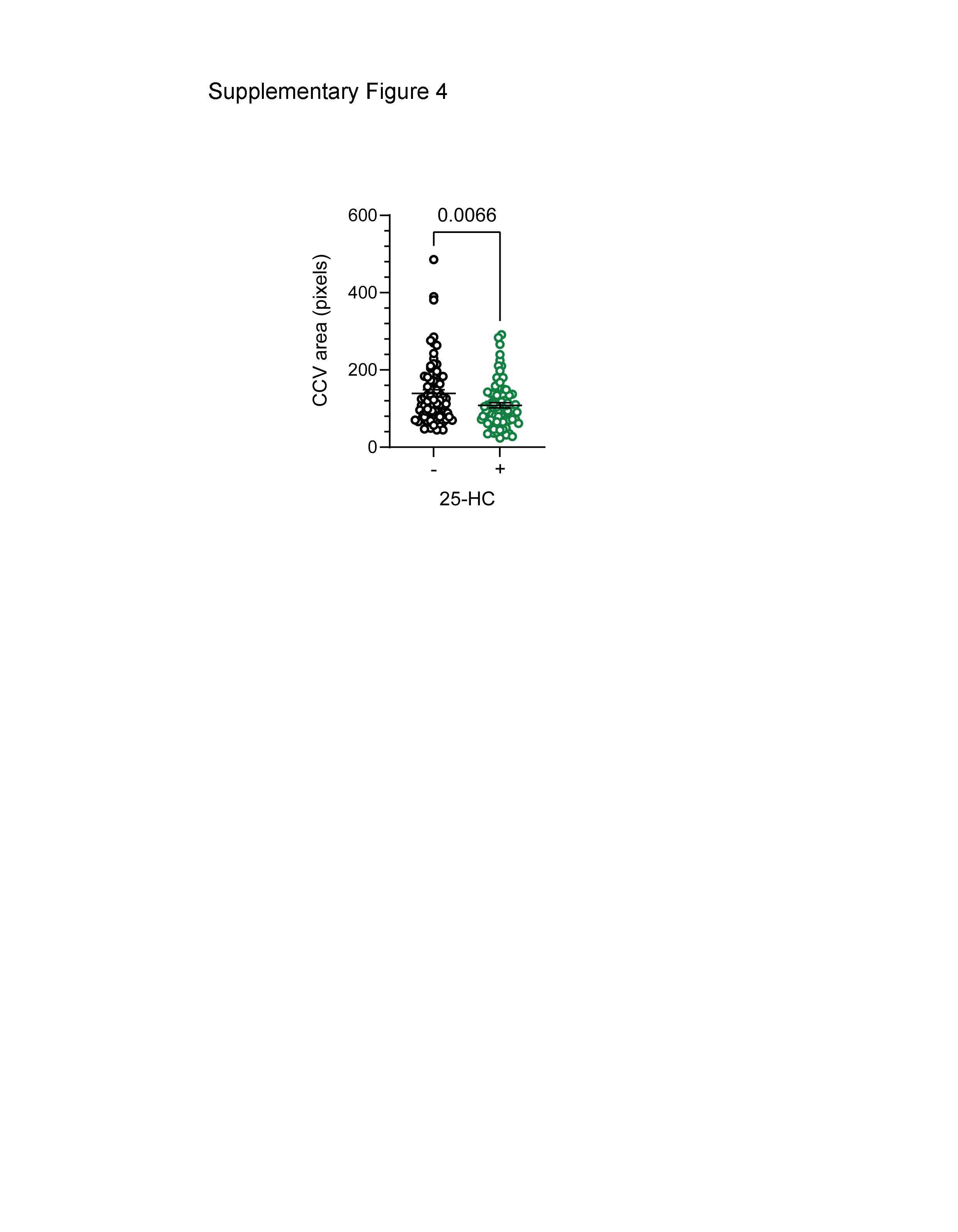

Supplement: FIG S4 [file mbio.03073-21-sf004.jpg]
